# Carboxypeptidase N1 is anticipated to be a synergy metrics for chemotherapy effectiveness and prognostic significance in invasive breast cancer

**DOI:** 10.1186/s12935-021-02256-5

**Published:** 2021-10-28

**Authors:** Ranliang Cui, Chaomin Wang, Tiantian Li, Jialei Hua, Ting Zhao, Li Ren, Yichao Wang, Yueguo Li

**Affiliations:** 1 Department of Clinical Laboratory, Tianjin Medical University Cancer Institute and Hospital, Tianjin’s Clinical Research Center for Cancer, Key Laboratory of Cancer Prevention and Therapy, National Clinical Research Center for Cancer, Huanhuxi Road, Hexi District, Tianjin, 300060 China; 2 Department of Clinical Laboratory Medicine, Taizhou Central Hospital (Taizhou University Hospital), No. 999 Donghai Road, Jiaojiang District, Taizhou, 318000 Zhejiang China; 3 School of Medical Laboratory, Tianjin Medical University, Tianjin, China

**Keywords:** Carboxypeptidase N1, CA153, Chemotherapeutic efficacy, Prognosis, Invasive breast cancer

## Abstract

**Background:**

The incidence and mortality of invasive breast cancer (IBC) are increasing annually. Hence, it is urgently needed to determine reliable biomarkers for not only monitoring curative effects, but evaluating prognosis. In present study, we aim to determine the potential role of Carboxypeptidase N1 (CPN1) in IBC tissues on chemotherapeutic efficacy and poor prognosis.

**Methods:**

The expression level of CPN1 in IBC tissue samples (n = 123) was quantified by tissue microarray technique and immunohistochemical staining. Moreover, sera of IBC patients (n = 34) that underwent three to five consecutive chemotherapy sessions were collected. The patients were randomly stratified into a training (n = 15) as well as a validation group (n = 19). The expression of serum CA153 and CPN1 was quantified by electrochemiluminescence and ELISA assay, respectively.

**Results:**

By univariate and multivariate Cox regression analysis, we show that CPN1 expression in IBC tissues, as an independent risk factor, is related to a poor overall survival (OS) and progression-free survival (PFS) (P < 0.05). Analysis of the data revealed that CPN1 over-expression could be consistently linked to adverse clinicopathological features such as lymph node metastasis and the pathological stage (pTNM) (P < 0.05). The serum CPN1 level trajectory of individual patients generally decreased during chemotherapy. In line with these findings were changes in the follow-up ultrasonography and a consistent decrease in serum CPN1 levels. The comparison of the area under the receiver operating curves (ROC) revealed that CPN1 has a better surveillance value than CA153 in the training (AUC_CPN1_ = 0.834 vs. AUC_CA153 _= 0.724) as well as the validation set (AUC_CPN1_ = 0.860 vs. AUC_CA153_ = 0.720) when comparing cycle2 versus cycle3.

**Conclusions:**

CPN1 is a suitable potential biomarker for chemotherapeutic surveillance purposes as well as being an appropriate prognostic indicator which would support an improved chemotherapy regimen.

**Supplementary Information:**

The online version contains supplementary material available at 10.1186/s12935-021-02256-5.

## Introduction

Invasive breast cancer (IBC) is one of the most prevalent malignancies in women, with high-incidence and quick-progression. The malignancy is due to its infiltration and metastasis of IBC are responsible for cancer-related deaths in women [1, 2]. In clinical practice, conventional management strategies for IBC comprise surgery, or surgery in combination with adjuvant chemotherapy. However, for an appropriate treatment, prior evaluation and staging of the disease are necessary that might be subject to an evaluation bias. The pathological and molecular phenotyping of breast cancer can serve as a basis for the treatment, but both are not suitable as concomitant surveillance indicators because of the invasive character of the disease. Furthermore, the available histopathological information is often incomplete and limited [3].

Surveillance indicators comprise radiological and serological markers that can be combined to detect the chemotherapeutic efficacy. Ultrasound are one of the primary modalities available for diagnosing breast cancer and are recommended screening tests for women at high risk for breast cancer [4]. For its non-radiological, and non-invasive nature, as well as its convenience in clinical application which cannot be substituted. Moreover, ultrasonic sensing can be complemented with individual serum marker that can conveniently be obtained from patients, in predicting disease progression as well as allow for the long term observation of treatment effects [5]. Serum CA15-3 is a macromolecular glycoprotein antigen that mainly exists in the luminal side of the normal mammary epithelium, and its concentration in serum increases dramatically when cells become malignant. CA15-3 is a great value biomarker to evaluate and predict for breast cancer [6]. Although current conventional breast cancer markers, such as CA153, play vital roles in the detection, its specificity, in the evaluation of disease development as well as in the continuous evaluation of treatment efficacy, does not fully clinical demands [7]. Recent studies have shown that alternative methods may also be valid to evaluate the therapeutic efficacy of breast cancer treatment, including the detection of circulating tumor cells (CTCs) [8, 9], circulating tumor DNA (ctDNA) [10] and exosomes [11] collected by liquid biopsy. While proven to yield the potential for prediction, their limitations in clinical practice include low concentrations in body fluids and lower sensitivity and specificity compared to traditional serum markers [12]. Therefore, it is of major importance to identify new markers for a complimentary diagnostic and therapy efficacy determination [13].

Previously, we used nanochip and mass spectrometric analysis methods to filter out C3f polypeptide fragments from the microenvironment of breast tumors and found that carboxypeptidase N (CPN) can hydrolyze the protein polypeptides produced by the C3f fragments and is specific to this enzyme [14]. And we have illustrated an ectopic expression of CPN in tumor tissue from patients and murine models of breast cancer. In present study, CPN was proposed as a new diagnostic marker for IBC [14]. CPN, also known as an arginine/lysine carboxypeptidase, kininase I, or anaphylatoxin inactivator, is a member of a larger family of zinc metallopeptidases. CPN is a tetramer composed of two identical catalytic (CPN1) and regulatory subunits (CPN2), with CPN1 being the main functional subunit of the holoenzyme [15]. Moreover, CPN2 has been disclosed as a method for determining breast cancer. In addition, CPN1 production in serum is correlated with tumor size, clinical stage, and metastasis, which indicated that CPN be used as an innovative tumor marker for ancillary diagnosis of IBC metastasis [16]. Data from a database showed that CPN1 is elevated in several specific types of breast cancer, compared with normal and other non-triple-negative breast cancers, triple-negative breast cancer has higher CPN1 mRNA expression. The database also showed that patients with high CPN1 levels and the pathological classification of basal-like, Estrogen Receptor (ER)-positive, luminal A, luminal B, and wild-type TP53 breast cancer had a shorter overall survival time and poorer prognosis. However, the extent to which CPN1 expression in tissues and serum is likely to be a marker for detecting the efficacy of IBC treatment remains to be investigated.

## Materials and methods

### Tissue and serum samples

The IBC tissue microarrays were purchased from Shanghai Core Ultra Biotechnology Co., Ltd (catalog number: HBreD140Su07), which contained a total of 140 cases of IBC cancer tissues collected from the patients. Patients were aged from 37 to 88 years old, operated from August 2004 to December 2008, and followed up until July 2014, with no lost cases or incomplete tissue core sites. With 17 cases of dissection during the histochemistry operation, a total of 123 patients were enrolled in the study.

From October 2020 to January 2021, 34 patients were enrolled from Tianjin Medical University Cancer Institute & Hospital, who were diagnosed with IBC by histopathology. The clinical characteristics of the subjects are summarized in Supplementary information (Additional file 1: Table S1). Chemotherapeutic efficacy during this study was based on imaging according to the "Response Evaluation Criteria in Solid Tumors (RECIST 1.1)" [17] with following criteria: (1) Complete response (CR): complete disappearance of all lesions; (2) Partial response (PR):decrease in target lesion burden by ≥ 30% from baseline; (3) Stable disease (SD): residual/persistent tumor lesions and neither PD nor PR has been achieved; (4) Progression of disease (PD): increase in target lesion burden by ≥ 20% from nadir and new lesion or PD based on non-target lesion. Venous serum samples from fasted subjects were collected and frozen in a − 20 ℃ freezer (SANYO MPR-215F Medicool).

### Immunohistochemistry

CPN1 expression of 123 IBC tissue samples were detected with immunohistochemical (IHC) assay. The experimental materials and procedures included CPN1 antibody (Proteintech, 13385-1-AP, Wuhan, China, concentration 1.3 μg/ml), broad-spectrum secondary antibody and DAB color development kit. The tissue paraffin blocks were stained by immunohistochemical DAB according to the steps of antigen repair, antigen–antibody reaction, color rendering, dehydration, sealing. Section images were obtained using a tissue analysis platform (SITUOLI, Tissue FAXS Viewer, Jiangsu). The scores were determined using the following criteria: CPN1 expression was recorded in five random fields (100× magnification) using a light microscope. Positive intensity: no color, yellow, brownish-yellow, brownish-brown, representing scores of 0, 1, 2, and 3, respectively. Percentage of positive cells: no positive cells, 1–25%, 26–50%, 51–75%, and > 75% represented 0, 1, 2, 3, and 4 points. The final staining scores were determined by multiplying the intensity scores by the staining extent and ranged from 0 to 12. IHC scores ≤ 4 were considered to indicate low levels of CPN1 expression, whereas scores from 5 to 12 were considered to indicate high levels of expression.

### ELISA for detecting serum CPN1

CPN1 was detected using a carboxypeptidase N1 enzyme linked immunosorbent assay kit (Wuhan Cloud-Clone Co., Ltd). The laboratory procedure was operated according to strict instructions. Dilution of standards in multiples of the instructions to 1000 pg/ml, 500 pg/ml, 250 pg/ml, 125 pg/ml, 62.5 pg/ml, 31.2 pg/ml, 15.6 pg/ml. The standard dilution (0 pg/ml) was used as blank wells. After adding the sample and incubating at 37 °C, the liquid was discarded, adding working solution A, B and TMB substrate solution in turn, and then washing the plate with an automatic plate washer (Tecan HYDROFLEX) before each addition of reagents, add the reagent and incubate, then add the termination solution and incubate. The sample Optical Density (OD value) of the solutions was read at 450 nm wavelengths with a enzyme marker (Thermo Multiskan FC) to calculate the concentration of CPN1 by the standard curve of ELISA.

### Determination of CA153 and CEA concentrations in serum

The concentration of CA153 and CEA in serum was measured using a Roche electrochemiluminescence automated immunoassay system (Roche Cobas 801). CA153, CEA and related buffer reagents were provided by Roche and operated in strict accordance with the specifications of the manufacturer's instructions. According to the positive threshold value provided by the reagent company, CA153 was positive when the concentration reached 25 U/ml; CEA was positive when the concentration reached 5 ng/ml.

### Statistical analysis

SPSS 23.0, GraphPad Prism 9.0.0, statistical software, Pathway Builder Tool 2.0 and Adobe Photoshop 2020 were used for data analysis and chart production. Normally distributed continuous quantitative data representative of experimental results of each group of clinical specimens were compared with independent sample T-tests; 2-sided *P* < 0.05 shows statistical significance. The Kaplan–Meier method was used to assess OS and PFS, and the difference between survival curves was tested using the log-rank test. Cox regression was used to evaluate the prognostic value of different indicators. The training set ROC curve was obtained from the decline rate of two consecutive chemotherapy cycles, and the validation set ROC curve based on the predicted values of the training set.

## Results

### Immunohistochemical and survival analysis of CPN1 expression in tissue

**Fig. 1:**
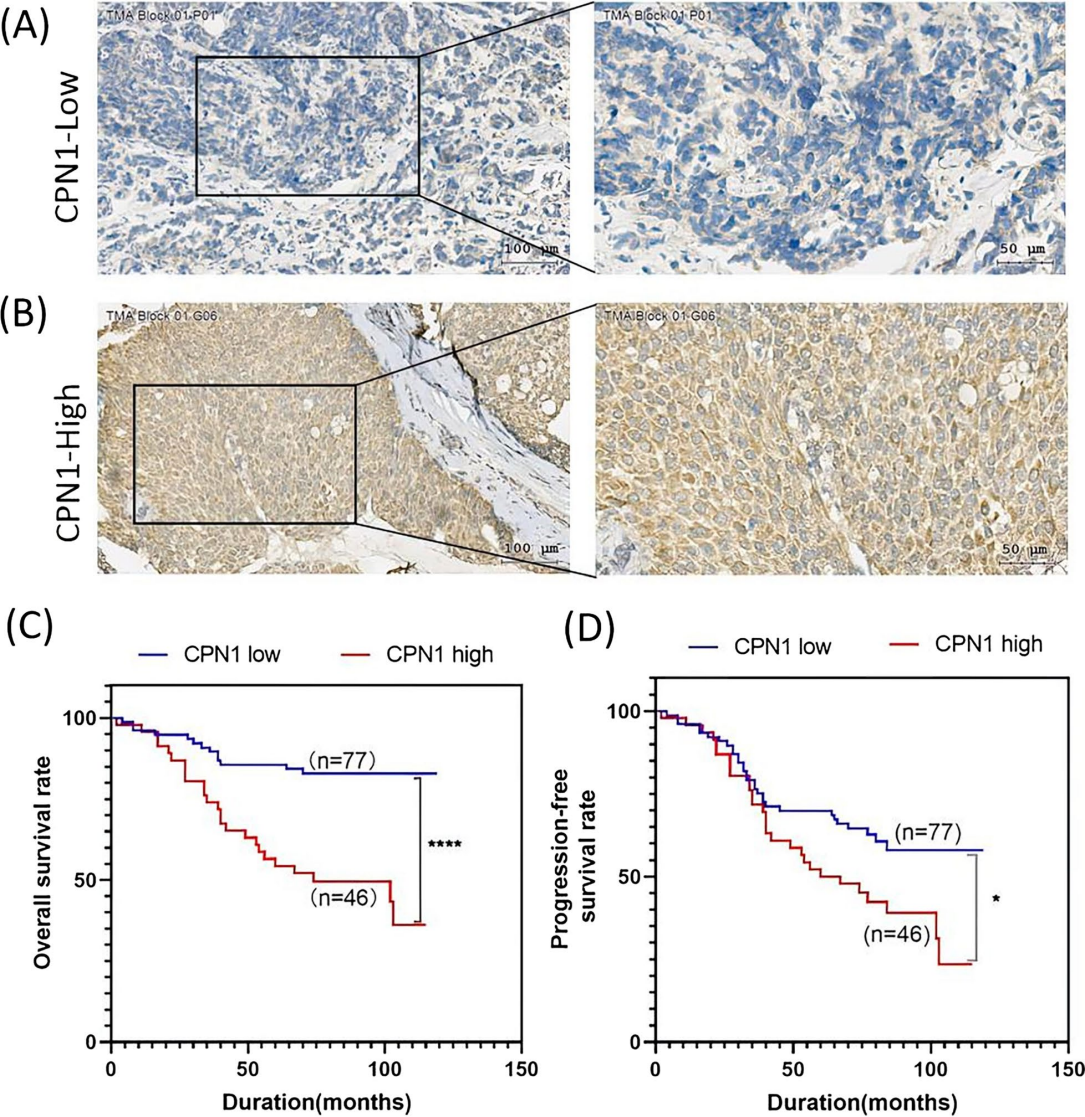
CPN1 expression in human IBC tissues associated with overall survival (OS) and Progression-free survival (PFS). **A**, **B** IBC tissues were stained with CPN1 by Immunohistochemistry (IHC). Representative IHC staining of CPN1-low group (**A**) and CPN1-high group (**B**). Tumoral CPN1 expression associated with overall survival (OS) and Progression-free survival (PFS). **C**, **D** Kaplan–Meier curves of OS (**C**) and PFS (**D**) in IBC patients according to the different levels of CPN1 based on the log-rank statistic test (*P < 0.05,****P < 0.001)

To investigate a potential correlation of CPN1 expression and IBC survival, IHC staining on tissue microarrays from 123 IBC patients was performed. The levels of CPN1 expression in IBC tissues showed a significant difference (Fig. 1A and B). Therefore, tissue samples were stratified into two groups with refer to CPN1 expression as follows: a CPN1-low group (IHC score ≤ 4, n = 77) and a CPN1-high group (IHC score > 4, n = 46). IBC patients with high CPN1 expression had a significantly poorer OS and PFS (*P* < 0.05) compared to patients with low CPN1 expression (Fig. 1C and D).The median OS was 64.9 and 80.5 months for the CPN1-low group and the CPN1-high group, respectively. The median PFS was 62.6 months in the CPN1-low group and 74.9 months in the CPN1-high group.

### CPN1 expression in IBC tissue and association with clinical variables

The data analysis revealed that CPN1 expression was positively correlated with TNM stage (*P* = 0.019) and lymph node metastasis (*P* = 0.006). Having shown this, we further analyzed the correlation between clinicopathologic parameters and patient outcomes by univariate analysis (Table 1). These results revealed that tumor size, lymph node metastasis and CPN1 status (all *P* < 0.05) were independent factors that affected OS (Table 2) and that were unfavourable predictors for a PFS (Additional file 1: Table S1).

**Table 1:** Correlation of tissue CPN1 expression with clinicopathological parameters

Parameters	Sample	CPN1		*x*^*2*^	*P* value
		Low	High		
Age (years) ≤ 60	77	53	24	2.851	0.091
> 60	46	23	23		
Tumor size (cm) ≤ 5	103	67	36	4.325	0.038*
> 5	20	9	11		
LN metastasis N0	61	38	23	3.031	0.082
N1	62	24	38		
TNM stage I–II	71	49	22	5.083	0.024*
III–IV	52	26	26		
Histological grade I–II	66	48	18	4.389	0.036*
III	57	32	45		
ER score ≤ 4	66	39	27	10.557	0.228
> 4	57	37	20		
PR score ≤ 4	91	58	33	12.553	0.128
> 4	32	18	14		
HER2 score ≤ 4	66	47	19	9.503	0.302
> 4	57	29	28		
KI67 score ≤ 4	94	57	37	10.8	0.213
> 4	29	19	10		
P53 score ≤ 4	77	47	30	7.97	0.436
> 4	46	29	17		

*Statistically significant (*P* < 0.05)

**Table 2:** Predictive factors of OS

Overall survival	Univariate analysis				Multivariate analysis		
	Cut off	HR	95% CI	*P* value	HR	95% CI	*P* value
Age (years)	60	1.053	0.552–2.008	0.875			
Tumor size (cm)	5	2.018	1.003–4.06	0.049*	0.751	0.205–2.753	0.666
T stage	3	1.696	1.081–2.662	0.022*	1.421	0.707–2.856	0.323
N stage	3	1.507	1.115–2.038	0.008*	2.18	0.875–5.433	0.094
Pathologic stage	3	1.02	0.751–1.384	0.612			
pT stage	3	2.3	1.332–3.971	0.003*	0.518	0.264–1.015	0.055
ER score	4	1.33	0.685–2.58	0.4			
PR score	4	0.4	0.156–1.023	0.047*	0.38	0.102–1.418	0.15
HER2 score	4	1.983	0.454–1.611	0.036*	0.508	0.254–1.154	0.51
KI67 score	4	0.825	0.293–2.323	0.716			
P53 score	4	1.024	0.541–1.939	0.475			
CPN1 score	4	3.438	1.784–6.624	< 0.001*	2.384	1.073–5.295	0.033*

HR: Cox proportional hazard ratio, 95% CI: 95% confidence interval

*Statistically significant (P < 0.05)

Subsequently, the multivariate analysis that was performed on CPN1 levels and the prognostic parameters found by univariate analysis, we identified independent predictors of OS and PFS. Our results revealed that CPN1 upregulation was a negative independent predictor for OS (HR = 3.275, 95% CI 2.201–4.873; *P* < 0.05) and PFS (HR = 2.309, 95% CI 1.164–4.580; *P* < 0.05) in patients with IBC.

### CPN1 dynamics during chemotherapy

**Fig. 2:**
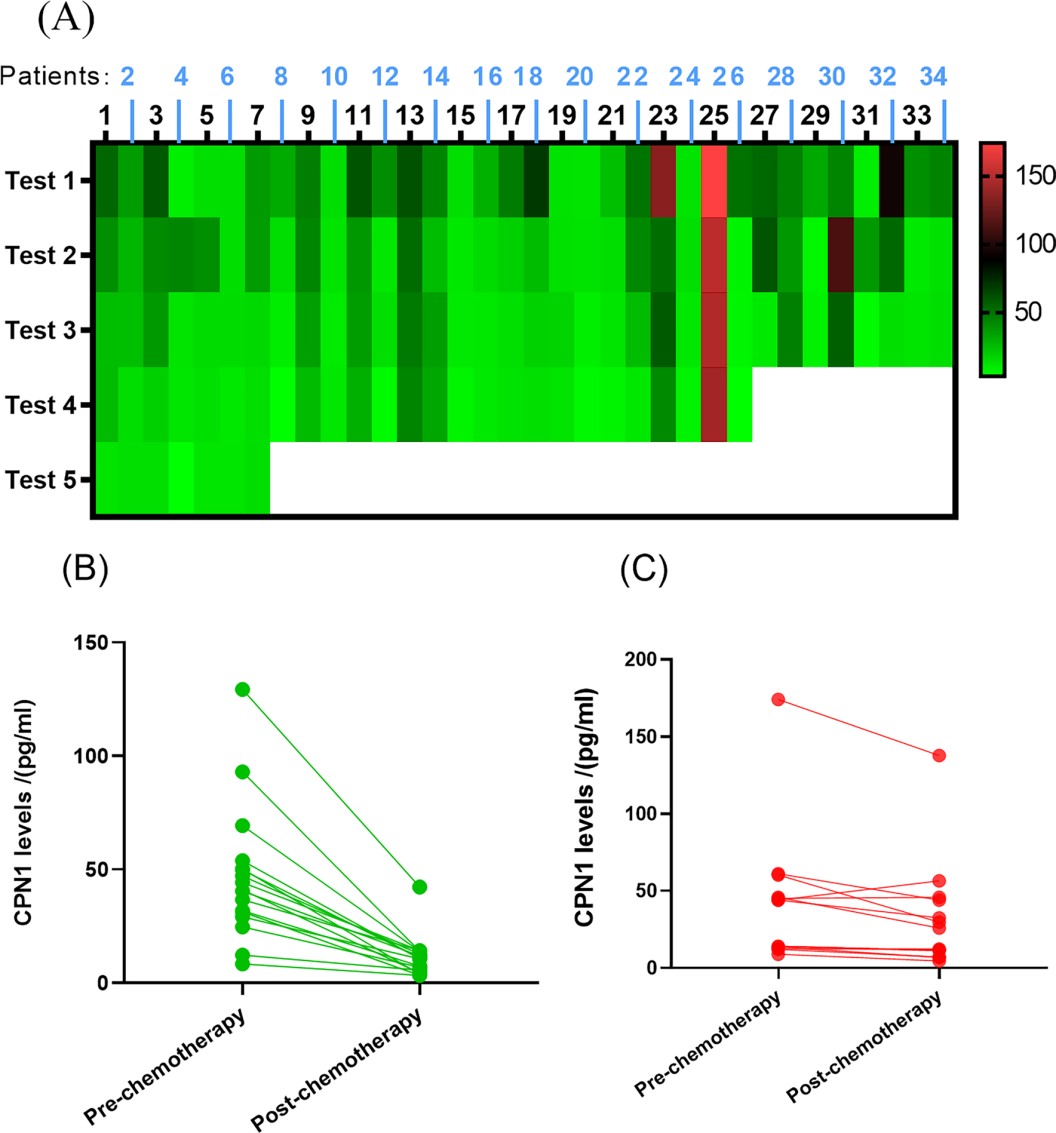
CPN1 detection and clinical performance. **A** Comparative analysis of serum CPN1 expression in 34 cases; **B**, **C** CPN1 levels before and after chemotherapy in breast cancer patients with **B** post-chemotherapy efficacy PR (n = 10), **C** post-chemotherapy efficacy SD (n = 24) in breast cancer patient cases before and after chemotherapy. *Test X: the X observation point

**Fig. 3:**
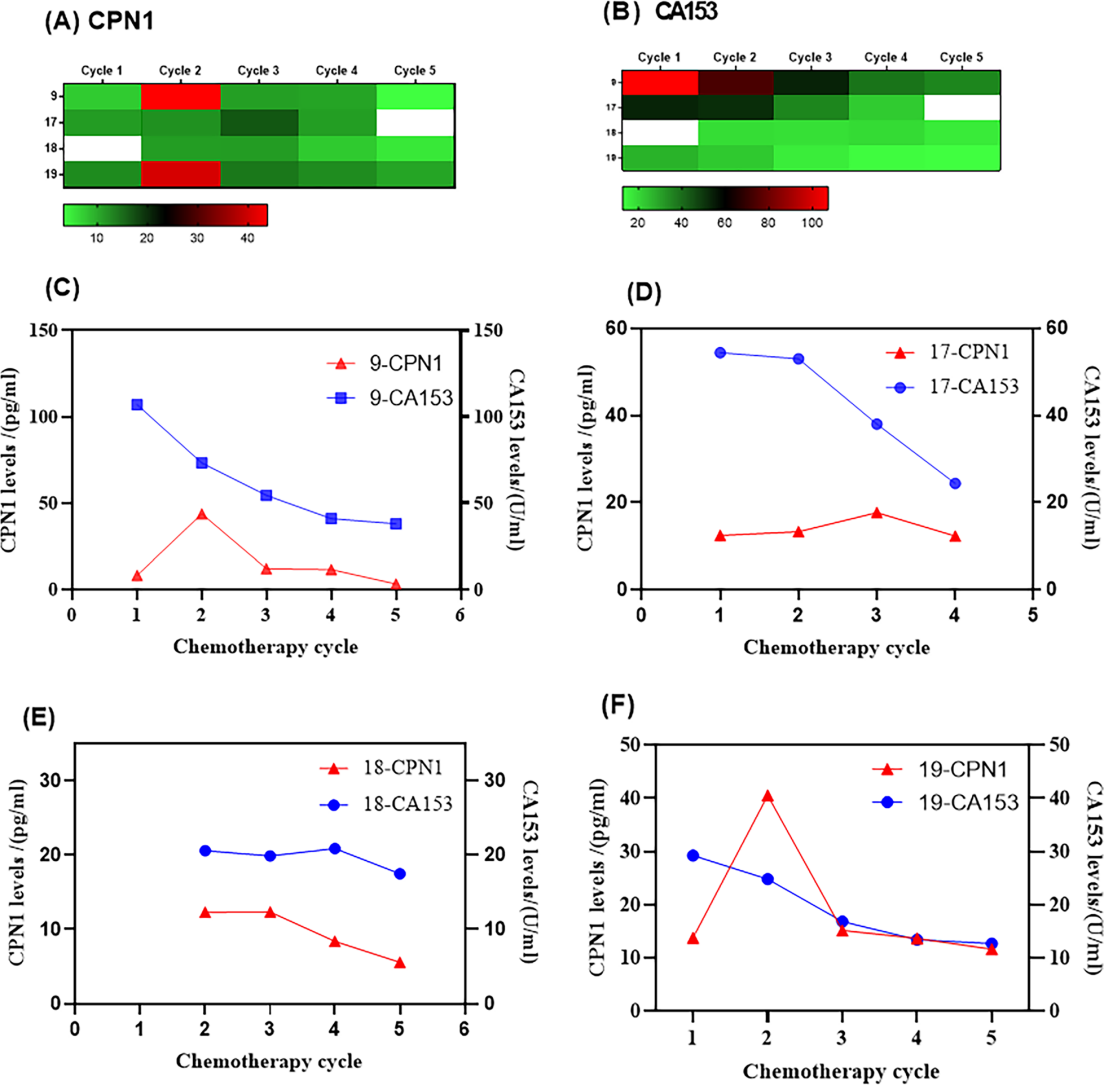
Serum CPN1 and CA153 levels in 4 chemotherapy-insensitive patients. **A** CPN1 levels; **B** CA153 levels; **C**–**F** Levels of CPN1 and CA153 in each chemotherapy-insensitive patient

In all 34 patients, Serum CPN1 levels tended to decrease with increasing chemotherapy cycles, with a significant decrease in PR patients (≥ 45% decrease), but not in SD patients (< 45% decrease) (Fig. 2). For comparison, CA153 and CEA levels variation in all cases (34) at five consecutive chemotherapy cycles (Additional file 1: Fig. S1). Compared to chemotherapy-sensitive patients, non-sensitive patients showed oscillatory expression of CPN1 when the chemotherapy regimen was changed (Fig. 3). Although CPN1 levels were initially high, they continued to decline after chemotherapy in clinical stage III + IV patients (Additional file 1: Fig. S2). In addition, the concentration of CPN1 fluctuated in patients with triple-negative breast cancer (TNBC) and in patients treated extensively with chemotherapy compared to CA153 (Additional file 1: Figs. S3, S4).

### Serum CPN1 as a potential surveillance biomarker for IBC

**Fig. 4:**
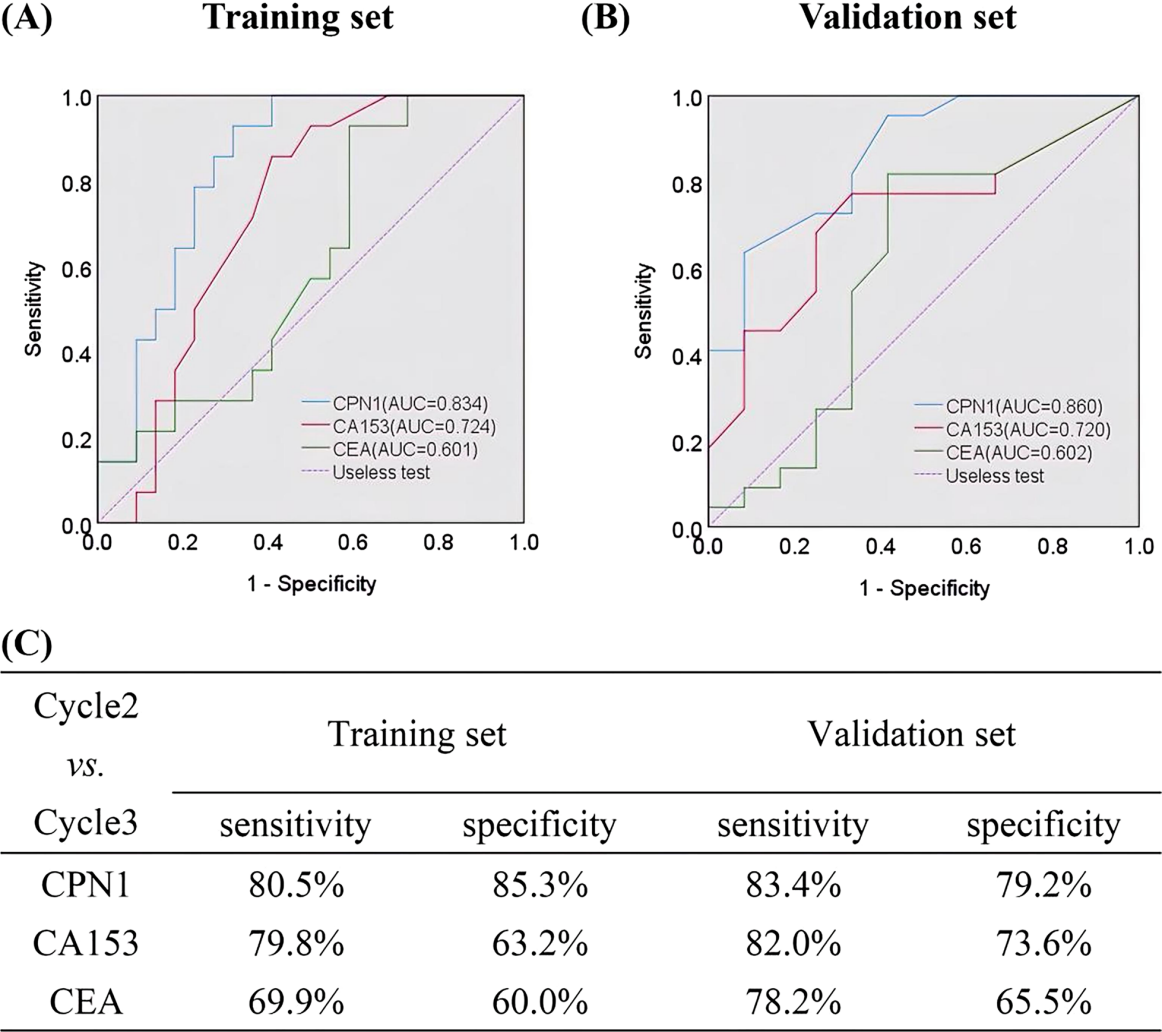
Serum CPN1 as a potential biomarker to predict chemotherapy effectiveness in IBC patients (Cycle2 vs*.* Cycle3). **A** Serum CPN1 as a potential biomarker to predict chemotherapy effectiveness compared with the level of CA153 and CEA by ROC analysis, the AUC of CPN1, CA153and CEA were 0.834, 0.724 and 0.601, *P* < 0.05. **B** Serum CPN1 as a potential biomarker to chemotherapy effectiveness compared with the level of CA153 and CEA by ROC analysis in the validation set. The AUC of CPN1, CA153 and CEA were 0.860, 0.720 and 0.602, *P* < 0.05. **C** The sensitivity and specificity of serum CPN1, CA153 and CEA for predicting chemotherapy effectiveness in the training set and validation set

CA153 and CEA are common biomarkers for IBC in clinical practice. Thus, we compared the suitability of using serum CPN1 levels and other markers as predictive tools for chemotherapy effectiveness. The ROC curve of CPN1 expression was better than those of CA153 or CEA in predicting chemotherapy effectiveness in both the training and validation set, respectively. (Fig. 4A, B). In a cycle2 versus cycle3 comparison of the training set, analysis of the sensitivity and specificity of these biomarkers showed that the sensitivity of CPN1 (80.5%) was higher than that of CA153 (79.8%) and that the specificity of the former was higher (85.3%) than that of the latter (63.2%). Moreover, the sensitivity and specificity of CPN1 (87.6% and 75.4%, respectively) were higher than that of CEA (65.0% and 50.3%, respectively) in predicting chemotherapy effectiveness. Equally, in the validation set, the sensitivity and specificity of CPN1 ((75.3% and 77.5%) were higher than that of CA153 (71.1% and 72.4%), respectively. Similar to the training set, the sensitivity and specificity of CPN1 (75.4% and 77.5%) were higher than that of CEA (62.6% and 65.8%) (Fig. 4C). Detailed information of a cycle3 versus cycle4 comparison follows (Additional file 1: Fig. S5).

### Comparison of radiology and CPN1 in typical cases

**Fig. 5:**
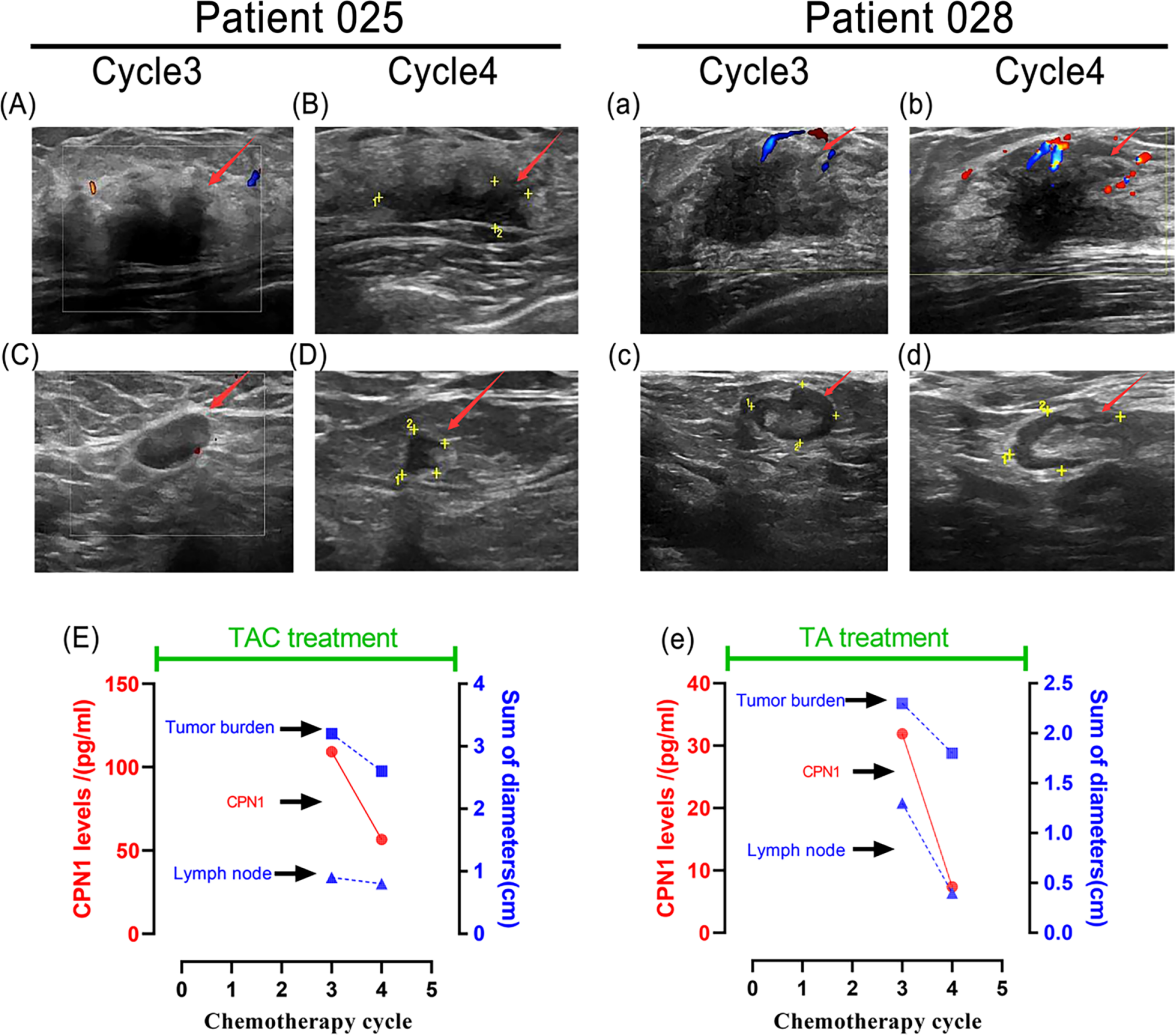
Representative patient cases. Patient025, a 53-year-old woman who diagnosed as TNBC and met PR criteria in cycle4. Patient028, a 53-year-old woman who diagnosed as HER2-positive breast cancer pathologically and met PR criteria in cycle4. **A**, **B**, **a**, **b** Left breast swelling, **C**, **D**, **c**, **d** Axillary lymph nodes; **E**, **e** Levels of CPN1 and tumor burden in Cycle3 and Cycle4

Two patients were compared in tracking sessions one who had a TAC treatment (patient 025) and one who had TA treatment (patient 028). The associated trajectories of serum CPN1 measurements and radiographic tumor burdens for these two typical patients are shown. The corresponding change in tumor and lymph node size was scaled down, with radiographic PR achieved at scan of cycle4. Patient 025 had a greater decline in CPN1 levels and tumor diameter (decline rate: 76.9%and 21.7%, respectively) than patient 028(decline rate: 48.2%and 18.8%, respectively). Longitudinal comparison of both patients revealed a better decrease rate of CPN1 than that of the tumor diameter, which may be more valuable for treatment efficacy determination (Fig. 5).

## Discussion

Our research revealed that the circulating peptides in the microenvironment of breast tumors which are specifically cleaved by CPN could be employed as peptide biomarkers for breast cancer using the "nanotrap" technology. We found that CPN was significantly over-expressed in breast cancer tissues. This was in line with the appearance of lymph node metastasis and corresponded to TMN staging in tissue microarrays [14]. Experiments with breast cancer cell lines indicated that metastatic cells over-expressed CPN and that reducing CPN expression leads to a suppression of the migratory and invasive properties of breast cancer cells [18]. CPN function is mainly determined by its catalytic subunit CPN1 [15]. Serology showed that CPN1 was expressed highly in both breast cancer and metastatic breast cancer and could be shown to be detected with a higher sensitivity and specificity than CA153 [16]. Therefore, we explored whether a drop in CPN1 levels would be a surveillance marker of chemotherapy efficiency and predicted a prolonged survival.

In this study, we examined the function of CPN1 in IBC and the results indicate that CPN1 is a negative indicator of OS and PFS in patients suffering from IBC.CPN1 over-expression was associated with poor clinicopathological features such as lymph node metastasis and the pathological stage (pTNM) (all *P* < 0.05). Our results showed that CPN1 overexpression could keep its prognostic value in predicting poorer survival (OS and PFS) in IBC. Consequently, CPN1 levels could be used as a potential prognostic biomarker for the stratification of breast cancer patients according to their risk.

We defined the significant declining trend as a ≥ 18% decrease in CPN1 during two consecutive chemotherapy cycles. We observed a pervasive descending tendency of CPN1 levels after chemotherapy, which is consistent with the assessment of the clinical efficacy for PR or SD. CPN1 levels significantly decreased in patients with PR (decline rate ≥ 45%) but not in patients with SD (decline rate < 45%). Additionally, we screened two individual typical cases of this study. Follow-up ultrasound revealed a reduction of tumor burden and downsizing of lymph nodes, which was corresponded to the trend of decreasing serum CPN1 levels. The actual drop in serum levels that we observed in patients who achieved a radiographic PR was much greater.

Based on the comparison of AUCs, it was found that the ROC curves of CPN1 were superior to CA153 and CEA in both the training and validation set, revealing that modeling was successfully established and outcomes were more reliable. It should be noted that CEA has poor sensitivity and specificity and can only be tested in combination with CA153 as a relevant marker for breast cancer. We compared CPN1 with CA153 or CEA alone and the results were in line with our expectations, with CEA having the lowest diagnostic value. The above results show that serum CPN1 levels reliable than other biomarkers (CA153 and CEA) in monitoring efficacy during chemotherapy although several cases of oscillatory expression of CPN1 during chemotherapy, including TNBC (3 cases), chemotherapy-insensitive (4 cases) and longer-term therapeutic (2 cases), have been detected.

TNBC is the breast cancer type with the worst prognosis owing to its highly heterogeneous and unspecific therapeutic target [19–23]. It has been shown that during chemotherapy, the breast masses enlarged and the CPN1 levels trended upwards. This trend could be abrogated by shortening the chemotherapy cycle after 3 cycles. It has been reported that dose-dense (DD) chemotherapy can minimize the re-growth of tumor cells by shortening the inter-treatment interval [24–28]. Therefore, the elevation of CPN1 levels may be suggestive of an alteration in the chemotherapy regimen for TNBC.

Resistance to chemotherapy during chemotherapy remains the main culprit for treatment failure for this deadly disease after surgery and radiation therapy [29, 30]. In this trial, oscillatory expression of CPN1 in the four chemotherapy-insensitive patients was characterized by a rise during chemotherapy cycle 2 or 3. A review of the records revealed a modification of major chemotherapy drugs (Paclitaxel and Adriamycin) and a diminution of the tumor and lymph node size. Paclitaxel is excreted extracellularly, leading to chemotherapy resistance mechanisms [31, 32]; Adriamycin promotes drug resistance mechanisms in neutrophil phenotypic polarization leading to tumor cell proliferation [33]. In parallel, CPN1 decreased and this was consistent with the prognosis which is why we speculated that the chemotherapy insensitivity was caused by chemoresistance. The specific mechanism is shown in Additional file 1: Figures S6A and S6B. Therefore, we hypothesize that CPN1 expression is suggestive of chemotherapy resistance.

Meanwhile, the study also found an irregular fluctuation of CPN1 in two patients with longer-term chemotherapy and the stable declining trend of CA153 in this time period. Inspection of the medical records revealed that alanine aminotransferase (ALT) and aspartate aminotransferase (AST) values significantly increased later (51U/L and 41U/L, respectively) compared to before (18U/L and 16U/L, respectively), which led us to assume that the patient might have experienced liver damage related to chemotherapy drugs [34, 35]. The detailed mechanism is shown that Mechanism of cytotoxic damage to hepatocytes during the metabolic transformation and excretion of chemotherapeutic drugs [36–39] (Additional file 1: Figure S6C). In addition, serum CA15-3 is a large glycoprotein antigen that is primarily present in the lumen of normal mammary epithelial cells, and its concentration in serum increases dramatically when the cells become malignant [6]. Human carboxypeptidase N (CPN), a member of the CPN/E subfamily of "regulatory" metallo-carboxypeptidases, is an extracellular glycoprotein synthesized in the liver and secreted into the blood [40]. Liver injury caused by certain chemotherapeutic drugs can lead to hepatocyte necrosis, resulting in a large release of CPN1 from hepatocytes. The prolongation of chemotherapy and long-term liver injury lead to a decrease in CPN1 synthesis and secretion into the blood by hepatocytes, and in summary, CPN1 showed a trend of first increasing and then decreasing. In contrast, CA153 does not exist in hepatocytes, therefore, it shows a decreasing trend during chemotherapy. Consequently, it is suspected that fluctuations in CPN1 levels might indicate liver injury, these will however require further mechanistic research (Additional file 2).

## Conclusions

In conclusion, this study is the first to explore the value of CPN1 for prognostic prediction and efficacy surveillance. The results indicate that CPN1 can be used as a biomarker for monitoring as well as prognosis in IBC. Patients with poor prognosis and chemotherapeutic efficacy, as identified based on CPN1 levels in the blood or tissue, could potentially benefit from CPN1-directed therapies in the future.

## Supplementary Information

**Additional file 1: **Supplementary materials and methods. **Table S1.** Predictive factors of PFS. **Table S2.** Characteristics of serum samples with invasive breast cancer (IBC). **Table S3** Expression of CPN1 and its correlation with clinicopathological parameters (Cycle2 vs. Cycle3)**.** The relationship between CPN1 and clinical features of breast cancer patients carried out a single factor analysis. **Table S4.** Multifactorial analysis table of serum CPN1 levels in breast cancer patients (Cycle2 vs. Cycle3). Tumor size and LN metastasis were associated with CPN1 decline rate (*p* < 0.05). **Table S5**. Expression of CPN1 and its correlation with clinicopathological parameters (Cycle3 vs. Cycle4). The relationship between CPN1 and clinical features of breast cancer patients carried out a single factor analysis. **Table S6.** Multifactorial analysis table of serum CPN1 levels in breast cancer patients (Cycle3 vs. Cycle4).Tumor size and LN metastasis were associated with CPN1 decline rate (*p* < 0.05). **Fig. S1.** Serum CA153 (A) and CEA(B) levels variation in 34 cases at five consecutive observation points. **Fig. S2.** Concentration of serum markers in clinical stage III + IV patients (A) CPN1; (B) CA153. **Fig. S3.** Concentration of serum markers in TNBC patients (A) CPN1; (B) Serum CA153. **Fig. S4.** Serum CPN1 and CA153 concentrations in longer-term chemotherapy patients. (A)(C) CPN1; (B)(D)Serum CA153. **Fig. S5.** Serum CPN1 as a potential biomarker to predict chemotherapy effectiveness in IBC patients (Cycle3 *vs.* Cycle4). (A) Serum CPN1 as a potential biomarker to predict chemotherapy effectiveness compared with the level of CA153 and CEA by ROC analysis, the AUC of CPN1, CA153and CEA were 0.806, 0.650 and 0.609, *P* < 0.05. (B) Serum CPN1 as a potential biomarker to chemotherapy effectiveness compared with the level of CA153 and CEA by ROC analysis in the validation set. The AUC of CPN1, CA153 and CEA were 0.805, 0.700 and 0.633, *P* < 0.05. (C) The sensitivity and specificity of serum CPN1, CA153 and CEA for predicting chemotherapy effectiveness in the training set and validation set. **Fig. S6.** Mechanisms of chemoresistance by adriamycin and paclitaxel and drug-induced toxic liver injury. (A) Paclitaxel is excreted extracellularly, leading to chemotherapy resistance mechanisms; (B) Adriamycin promotes drug resistance mechanisms in neutrophil phenotypic polarization leading to tumor cell proliferation. (C) Mechanism of cytotoxic damage to hepatocytes during the metabolic transformation and excretion of chemotherapeutic drugs.

**Additional file 2.** Immunohistochemical images of all tissue specimens.

## Data Availability

The data that support the findings of this study will be made available from the corresponding authors upon reasonable request.

## Abbreviations

ALT: Alanine aminotransferase
AST: Aspartate aminotransferase
CA153: Carbohydrate antigen 153
CEA: Carcino-embryonic antigen
CPN1: Carboxypeptidase N1
CR: Complete response
CTC: Circulating tumor cell
DD: Dose-dense
ER: Estrogen receptor
HER-2: Human epidermal growth factor receptor-2
IBC: Invasion breast cancer
IHC: Immunohistochemistry
MDR: Multidrug resistance
NAC: Neoadjuvant or primary chemotherapy
OS: Overall survival
PD: Progression disease
PFS: Progression free survival
PR: Progesterone receptor
SD: Stable disease
TNBC: Triple-negative breast cancers
