# Supplementary material for: Carboxypeptidase N1 is anticipated to be a synergy metrics for chemotherapy effectiveness and prognostic significance in invasive breast cancer
Source: Cancer Cell Int. 2021 Oct 28;21:571. doi: 10.1186/s12935-021-02256-5 (PMC8555242; doi:10.1186/s12935-021-02256-5)
Supplement: Supplementary file 1 — Additional file 1: Supplementary materials and methods. Table S1. Predictive factors of PFS. Table S2. Characteristics of serum samples with invasive breast cancer (IBC). Table S3 Expression of CPN1 and its correlation with clinicopathological parameters (Cycle2 vs. Cycle3). The relationship between CPN1 and clinical features of breast cancer patients carried out a single factor analysis. Table S4. Multifactorial analysis table of serum CPN1 levels in breast cancer patients (Cycle2 vs. Cycle3). Tumor size and LN metastasis were associated with CPN1 decline rate (p < 0.05). Table S5. Expression of CPN1 and its correlation with clinicopathological parameters (Cycle3 vs. Cycle4). The relationship between CPN1 and clinical features of breast cancer patients carried out a single factor analysis. Table S6. Multifactorial analysis table of serum CPN1 levels in breast cancer patients (Cycle3 vs. Cycle4).Tumor size and LN metastasis were associated with CPN1 decline rate (p < 0.05). Fig. S1. Serum CA153 (A) and CEA(B) levels variation in 34 cases at five consecutive observation points. Fig. S2. Concentration of serum markers in clinical stage III + IV patients (A) CPN1; (B) CA153. Fig. S3. Concentration of serum markers in TNBC patients (A) CPN1; (B) Serum CA153. Fig. S4. Serum CPN1 and CA153 concentrations in longer-term chemotherapy patients. (A)(C) CPN1; (B)(D)Serum CA153. Fig. S5. Serum CPN1 as a potential biomarker to predict chemotherapy effectiveness in IBC patients (Cycle3 vs. Cycle4). (A) Serum CPN1 as a potential biomarker to predict chemotherapy effectiveness compared with the level of CA153 and CEA by ROC analysis, the AUC of CPN1, CA153and CEA were 0.806, 0.650 and 0.609, P < 0.05. (B) Serum CPN1 as a potential biomarker to chemotherapy effectiveness compared with the level of CA153 and CEA by ROC analysis in the validation set. The AUC of CPN1, CA153 and CEA were 0.805, 0.700 and 0.633, P < 0.05. (C) The sensitivity and specificity of serum CPN1, CA153 and CEA [file 12935_2021_2256_MOESM1_ESM.doc]

**Carboxypeptidase N1 is anticipated to be a synergy metrics for chemotherapy effectiveness and prognostic significance in invasive breast cancer**

**A table of contents**

**Contents**

**Supplementary Materials & Methods**

**Supplementary Table**

**Supplementary Figure**

**Supplementary Materials & Methods**

***Immunohistochemistry***

123 IBC tissue samples were used for immunohistochemical (IHC) analysis of CPN1 expression.The experimental materials and procedures included CPN1 antibody (PROTEINTECH,13385-1-AP,CHINA,concentration 1.3μg/ml), broad-spectrum secondary antibody and DAB color development kit.The tissue paraffin blocks were stained by immunohistochemical DAB according to the steps of antigen repair, antigen-antibody reaction, color rendering, dehydration, sealing.Section images were obtained using a tissue analysis platform (Tissue FAXS Viewer, SITUOLI).

The scores were determined using the following criteria: CPN1 expression was recorded in five random fields (100 × magnification) using a light microscope. Positive intensity: no color, yellow, brownish-yellow, brownish-brown, representing scores of 0, 1, 2, and 3, respectively. Percentage of positive cells: no positive cells, 1%-25%, 26%-50%, 51%-75%, and >75% represented 0, 1, 2, 3, and 4 points. The final staining scores were determined by multiplying the intensity scores by the staining extent and ranged from 0 to 12. IHC scores ≤4 were considered to indicate low levels of CPN1 expression, whereas scores from 5 to 12 were considered to indicate high levels of expression.

***ELISA assay***

CPN1 was detected using a carboxypeptidase N1 enzymelinked immunosorbent assay kit (Wuhan Cloud-Clone Co., Ltd). The laboratory procedure was operated according to strict instructions . Dilution of standards in multiples of the instructions to 1000pg/ml，500pg/ml，250pg/ml，125pg/ml，62.5pg/ml，31.2pg/ml，15.6pg/ml. The standard dilution (0pg/ml) was used as blank wells. After adding the sample and incubating at 37 degrees, discard the liquid, add working solution A, B and TMB substrate solution in turn, wash the plate with an automatic plate washer (Tecan HYDROFLEX) before each addition of reagents, add the reagent and incubate, then add the termination solution and incubate. The sample Optical Density of the solutions was read at 450nm wavelengths with a enzyme marker(Thermo Multiskan FC) to calculate the concentration of CPN1 by the standard curve of ELISA.

***Electrochemiluminescence***

The concentration of CA153 and CEA in serum was measured using a Roche electrochemiluminescence automated immunoassay system (Roche Cobas 801). CA153, CEA and related buffer reagents were provided by Roche and operated in strict accordance with the specifications of the manufacturer's instructions.

***Statistical methods***

Single-factor analysis of variance was used by ANOVA.Multi-factoranalysis of variance was used by logistic regression analysis. Data were presented as the mean ± SEM.

**Table S1 Predictive factors of PFS**

| Progression-free  survival | Univariate Analysis | | | | Multivariate Analysis | | |
| --- | --- | --- | --- | --- | --- | --- | --- |
|  | Cut off | HR | 95% CI | *P* value | HR | 95% CI | *P* value |
| Age(years) | 60 | 0.978 | 0.513-1.865 | 0.947 |  |  |  |
| Tumor size(cm) | 5 | 0.834 | 0.289-2.406 | 0.737 |  |  |  |
| T stage | 3 | 1.5 | 0.997-2.257 | 0.052 |  |  |  |
| N stage | 3 | 1.676 | 1.251-2.245 | 0.001* | 1.91 | 0.77-4.741 | 0.163 |
| Pathologic stage | 3 | 0.8 | 0.522-1.226 | 0.306 |  |  |  |
| pT stage | 3 | 1.796 | 1.087-2.964 | 0.022* | 0.638 | 0.246-1.651 | 0.354 |
| ER score | 4 | 1.276 | 0.657-2.477 | 0.472 |  |  |  |
| PR score | 4 | 0.412 | 0.161-1.054 | 0.064 |  |  |  |
| HER2 score | 4 | 2.081 | 1.097-3.947 | 0.025* | 1.347 | 0.127-4.88 | 0.123 |
| KI67 score | 4 | 0.875 | 0.311-2.464 | 0.801 |  |  |  |
| P53 score | 4 | 1.045 | 0.552-1.979 | 0.893 |  |  |  |
| CPN1 score | 4 | 3.075 | 1.597-5.920 | 0.001* | 2.309 | 1.164-4.580 | 0.017* |

Note:HR=Cox proportional hazard ratio, 95%CI=95% confdence interval,

*Statistically significant (*P*＜0.05).

**Table S2 Characteristics of serum samples with invasive breast cancer(IBC).**

|  | Training set (n=15) | Validation set (n=19) | Tatol (n=34) |
| --- | --- | --- | --- |
| Age(Mean(SD)) | 49.87(11.13) | 51.37(10.00) | 50.70(10.54) |
| Age(Median) | 54[32,67] | 51[32,67] | 53[32,67] |
| Size≤5cm | 10（66.7%） | 12（63.2%） | 22（64.7%） |
| Size＞5cm | 5（33.3%） | 7（36.8%） | 12（35.3%） |
| Pathology typing | NA | NA | NA |
| TNBC | NA | 4（31.6%） | 4（11.8%） |
| LuminalA | 4（26.7%) | 2（10.5%） | 6（17.6%） |
| LuminalB | 10（66.7%） | 8（42.1%） | 18（52.9%） |
| HER2-Positive | 1（6.7%） | 5（26.3%） | 6（17.6%） |
| Clinical staging | NA | NA | NA |
| Stage I | 1（6.7%） | 1（5.3%） | 2（5.9%） |
| Stage IIA | 1（6.7%） | 1（5.3%） | 2（5.9%） |
| Stage IIB | 6（40.0%） | 4（31.6%） | 10（29.4%） |
| Stage IIIA | 1（6.7%） | 5（26.3%） | 6（17.6%） |
| Stage IIIB | NA | 2（10.5%） | 2（5.9%） |
| Stage IIIC | 2（13.3%） | 3（15.8%） | 5（14.7%） |
| Stage IV | 4（26.7%) | 3（15.8%） | 7（20.6%） |
| Chemotheraphy regimen | NA | NA | NA |
| TE | 2（13.3%） | 3（15.8%） | 5（14.7%） |
| AC | 3（20.0%） | 3（15.8%） | 6（17.6%） |
| AT | 1（6.7%） | 1（5.3%） | 2（5.9%） |
| TCbPH | 1（6.7%） | 4（31.6%） | 5（14.7%） |
| TP | 3（20.0%） | 2（10.5%） | 5（14.7%） |
| TAC | 1（6.7%） | 2（10.5%） | 3（8.8%） |
| Double target joint | 1（6.7%） | 3（15.8%） | 4（11.8%） |
| PTX | 1（6.7%） | NA | 1（2.9%） |
| TX | 1（6.7%） | NA | 1（2.9%） |
| EC | NA | 1（5.3%） | 1（2.9%） |
| TC | 1（6.7%） | NA | 1（2.9%） |
| Lymph node metastsis | NA | NA | NA |
| N0 | 3（20.0%） | 1（5.3%） | 4（11.8%） |
| N1 | 6（40.0%） | 7（36.8%） | 13（38.2%） |
| N2 | 3（20.0%） | 8（42.1%） | 11（32.4%） |
| N3 | 3（20.0%） | 3（15.8%） | 6（17.6%） |
| Distant metastsis | NA | NA | NA |
| M0 | 11（73.3%） | 14（73.7%） | 25（73.5%） |
| M1 | 4（26.7%） | 5（26.3%） | 9（26.5%） |

Note:AC=Doxorubicin+Cyclophosphamide;AT=Epirubicin+Paclitaxe; EC=Epirubicin+Cyclophosphamide; TAC=Cyclophosphamide+Doxorubicin+Docetaxe; TC=Docetaxe+Cyclophosphamide; TP=Cisplatin+Paclitaxel; TX=Xeloda+Docetaxe; TE=Docetaxe+Epirubicin; PTX=Paclitaxel Injection.


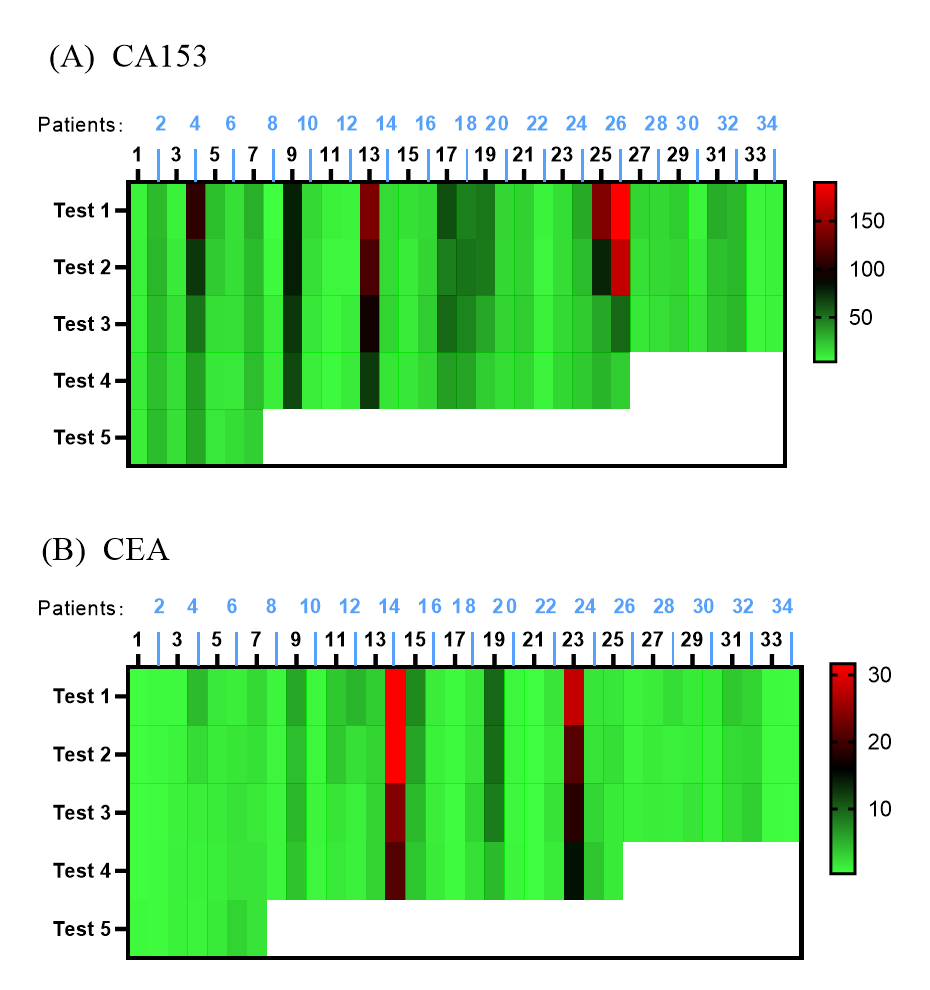


**Fig. S1 Serum CA153(A) and CEA(B) levels variation in 34 cases at five consecutive observation points**


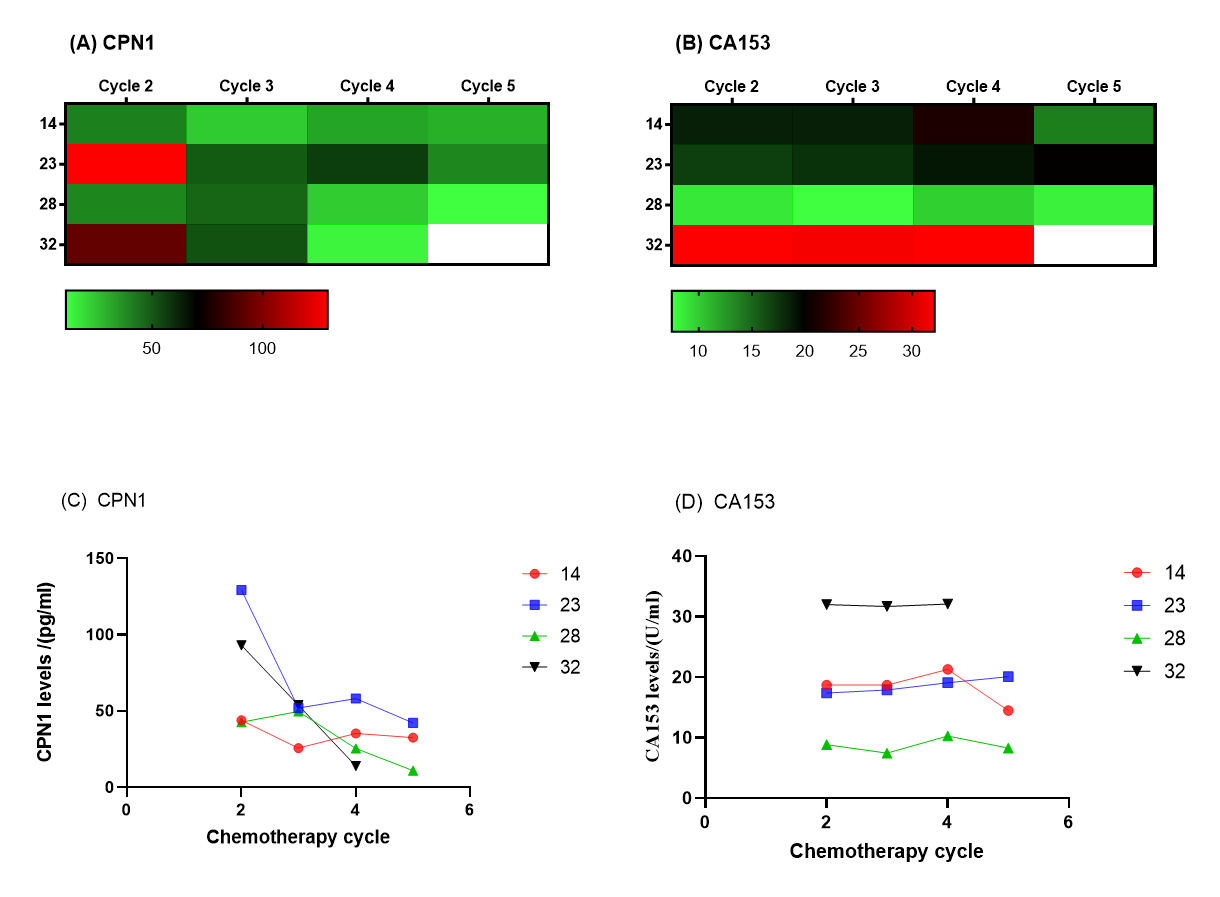


**Fig S2 Concentrations variation of CPN1 and CA153 in Patients 14,23,28,32 (Stage III+IV) (A)（C）CPN1 leves variation (Heatmap and line charts, respectively) (B); (D) CA153 levels variation (Heatmap and line charts, respectively)**


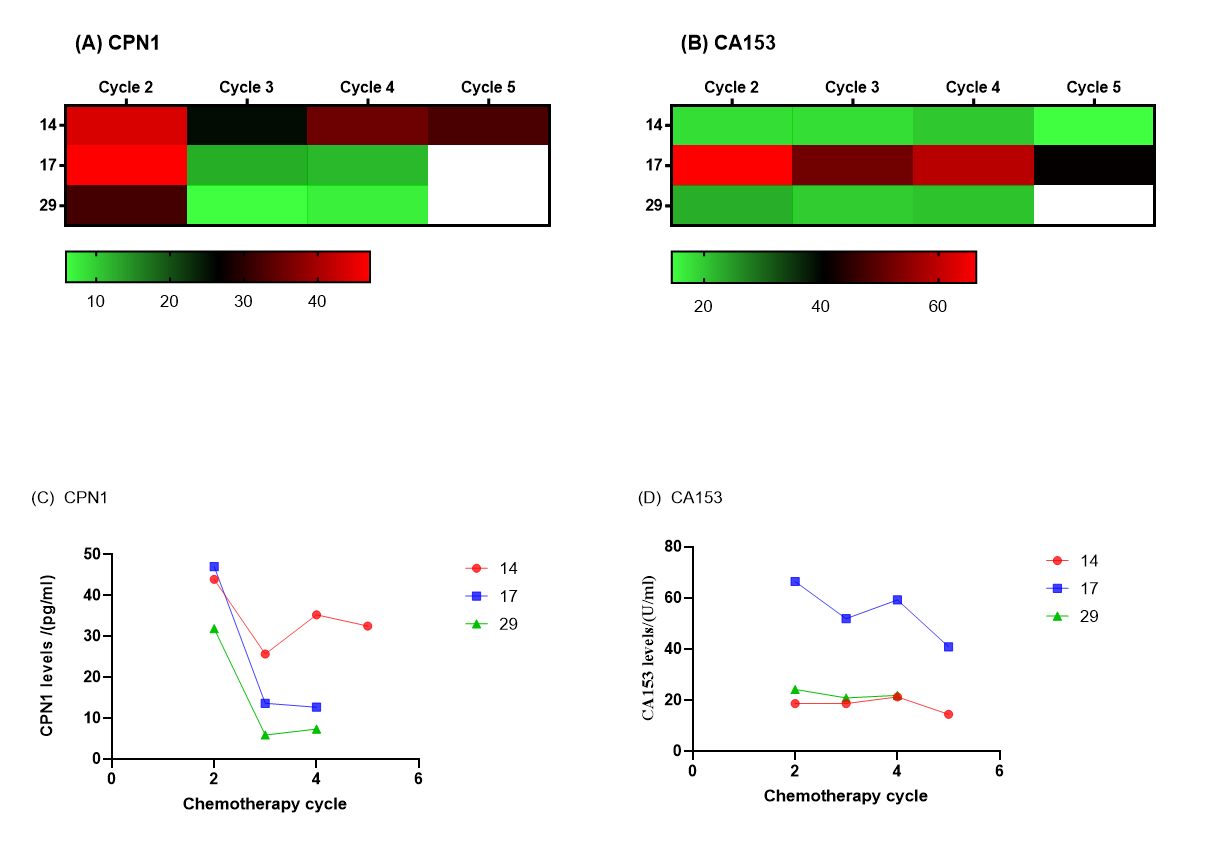


**Fig. S3 Concentrations variation of CPN1 and CA153 in patients14,17,29 (TNBC )** (A)（C）CPN1 leves variation (Heatmap and line charts, respectively) (B); (D) CA153 levels variation (Heatmap and line charts, respectively)

**Fig S4 concentrations variation of CPN1 and CA153 in patients 2,5,16,29(longer-term chemotherapy).** (A)（C）CPN1 leves variation (Heatmap and line charts, respectively) (B); (D) CA153 levels variation (Heatmap and line charts, respectively)


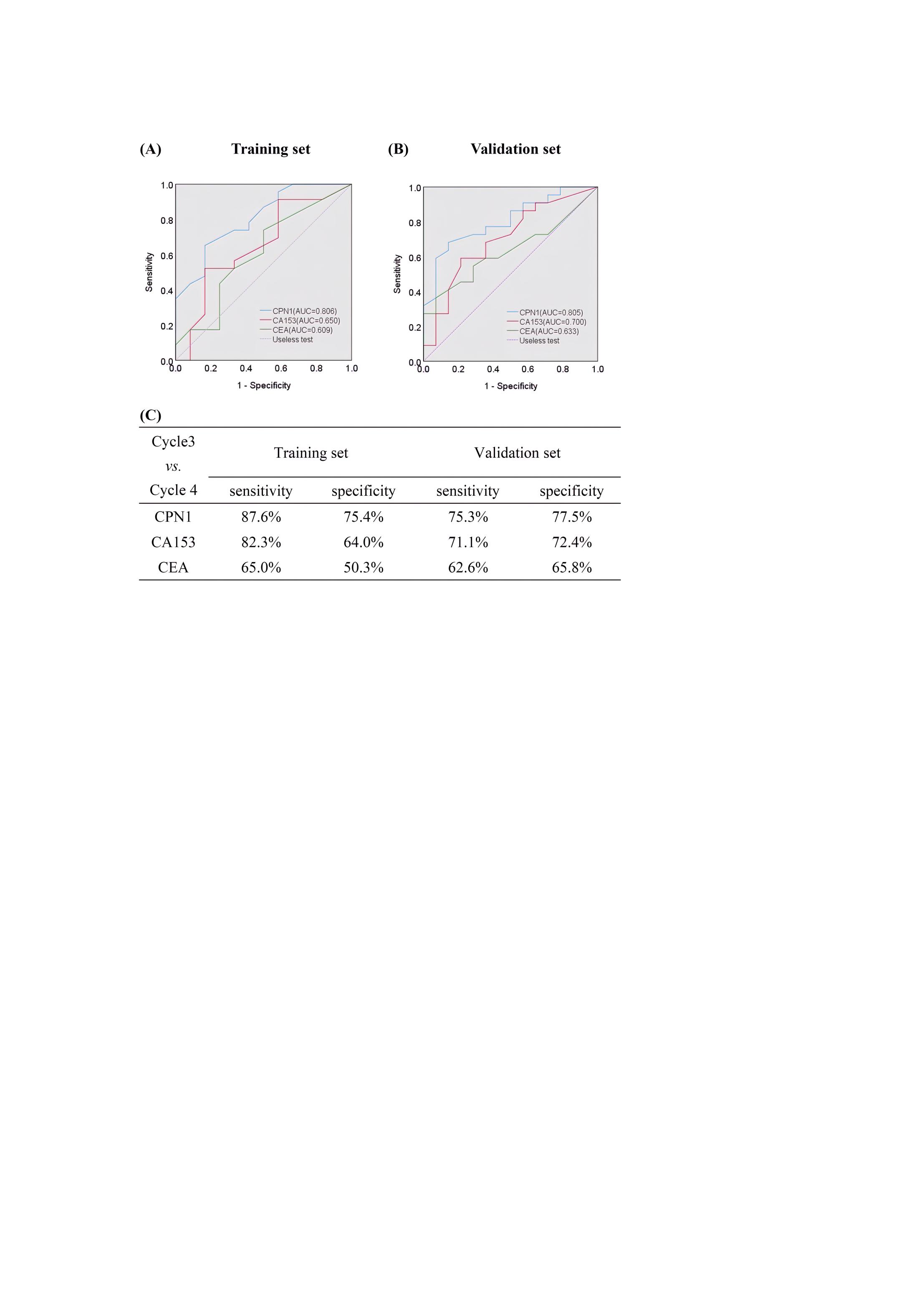


**Fig. S5 Serum CPN1 as a potential biomarker to predict chemotherapy effectiveness in IBC patients(Cycle3 *vs.* Cycle4).** (A) Serum CPN1 as a potential biomarker to predict chemotherapy effectiveness compared with the level of CA153 and CEA by ROC analysis, the AUC of CPN1, CA153and CEA were 0.806, 0.650 and 0.609, *P*<0.05. (B) Serum CPN1 as a potential biomarker to chemotherapy effectiveness compared with the level of CA153 and CEA by ROC analysis in the validation set. The AUC of CPN1, CA153 and CEA were 0.805, 0.700 and 0.633, *P*<0.05.(C) The sensitivity and specificity of serum CPN1, CA153 and CEA for predicting chemotherapy effectiveness in the training set and validation set.

**
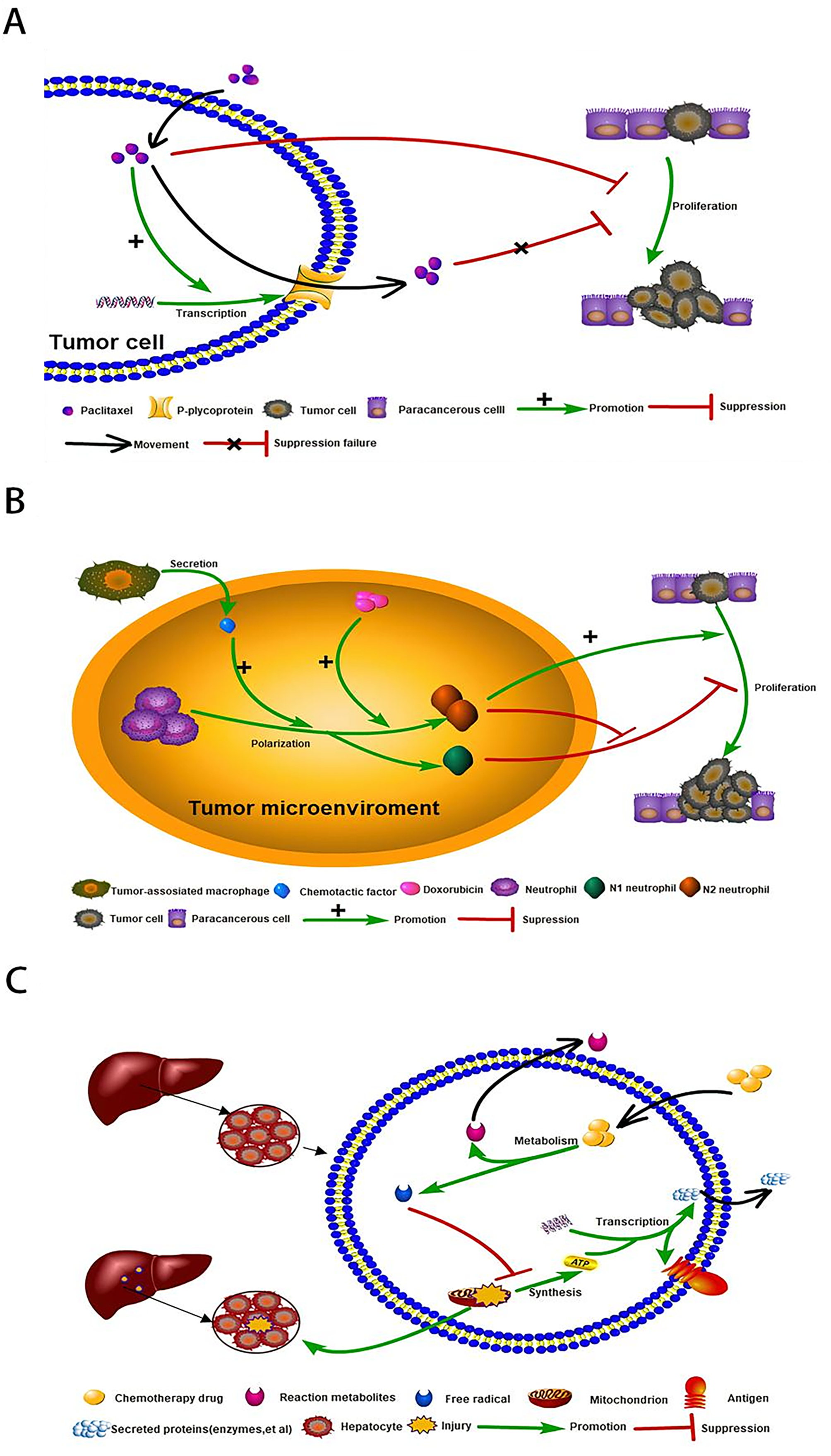
**

**Fig. S6** **Mechanisms of chemoresistance by adriamycin and paclitaxel and drug-induced toxic liver injury.**(A) Paclitaxel is excreted extracellularly, leading to chemotherapy resistance mechanisms;(B)Adriamycin promotes drug resistance mechanisms in neutrophil phenotypic polarization leading to tumor cell proliferation .(C)Mechanism of cytotoxic damage to hepatocytes during the metabolic transformation and excretion of chemotherapeutic drugs .
