# Supplementary material for: Carboxypeptidase N1 is anticipated to be a synergy metrics for chemotherapy effectiveness and prognostic significance in invasive breast cancer
Source: Cancer Cell Int. 2021 Oct 28;21:571. doi: 10.1186/s12935-021-02256-5 (PMC8555242; doi:10.1186/s12935-021-02256-5)
Supplement: Supplementary file 2 — Additional file 2. Immunohistochemical images of all tissue specimens. [file 12935_2021_2256_MOESM2_ESM.pdf]

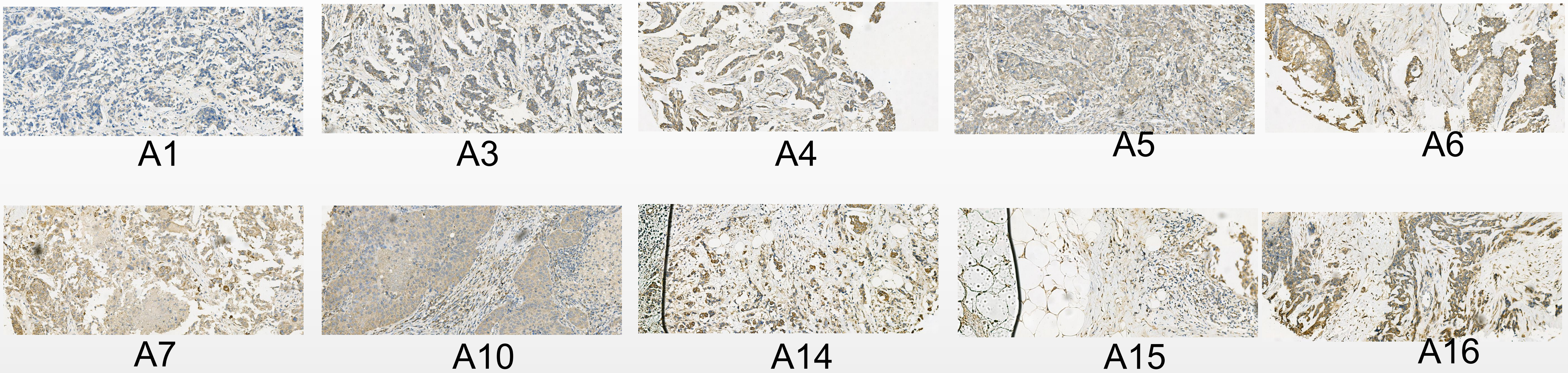
A1
A3
A4
A5
A6
A7
A10
A14
A15
A16

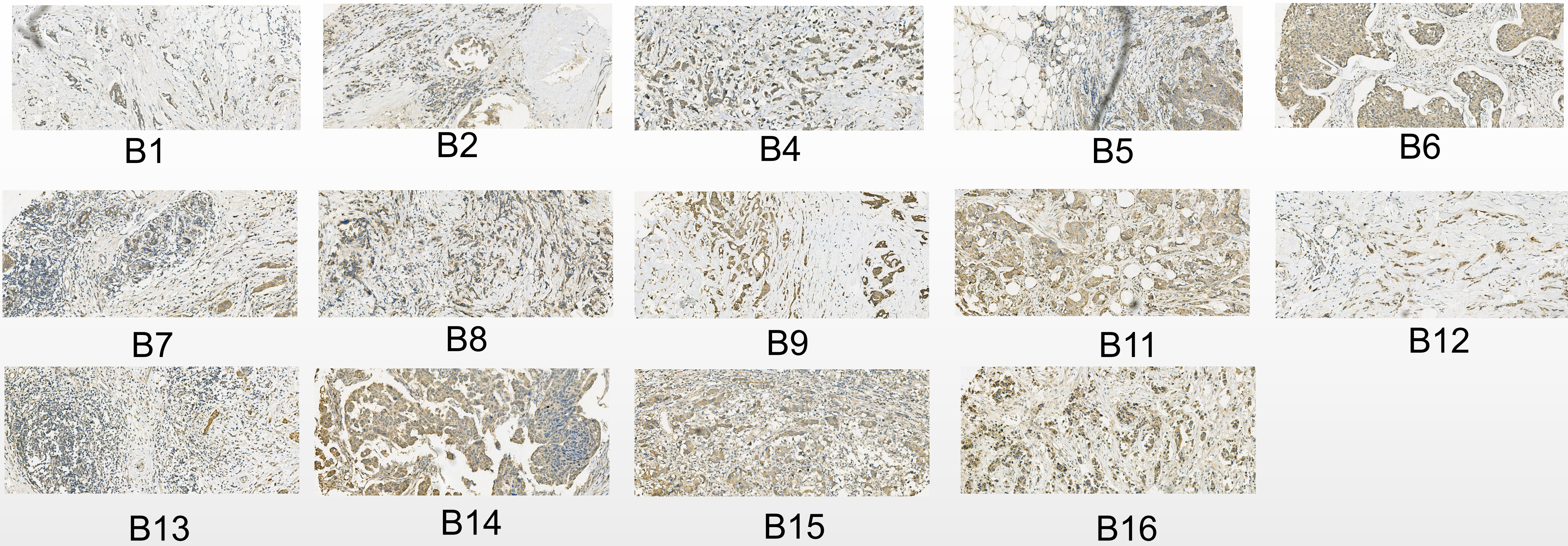
B1
B2
B4
B5
B6
B7
B8
B9
B11
B12
B13
B14
B15
B16

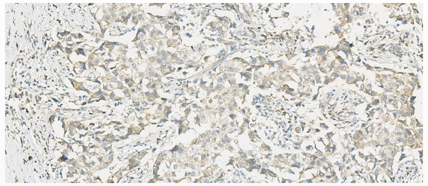

C1

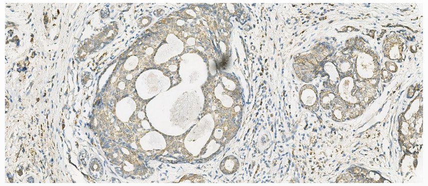

C2

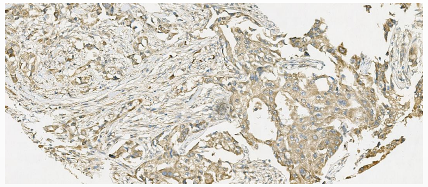

C3

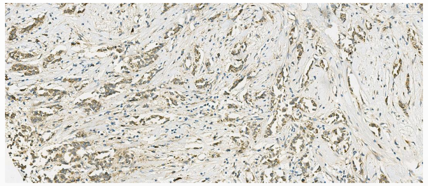

C4

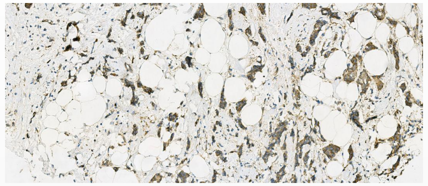

C5

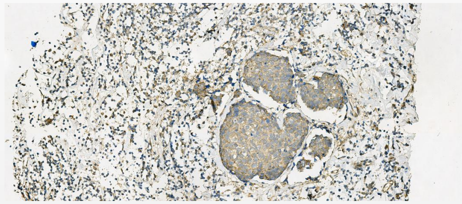

C6

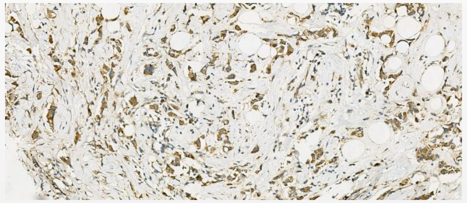

C9

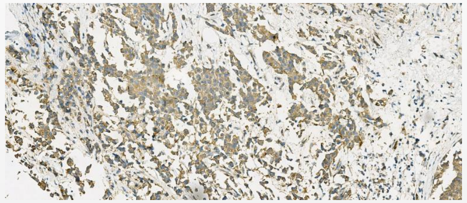

C10

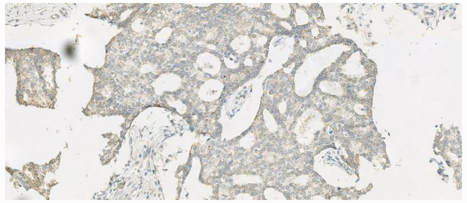
D1
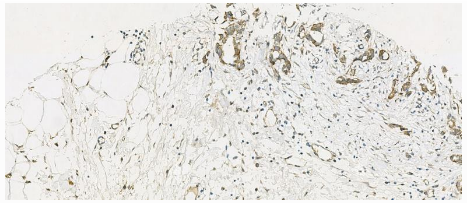
D2
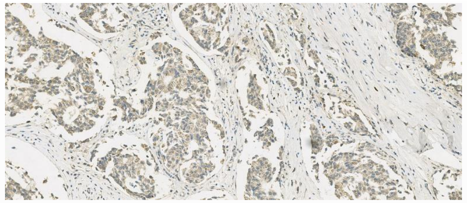
D3
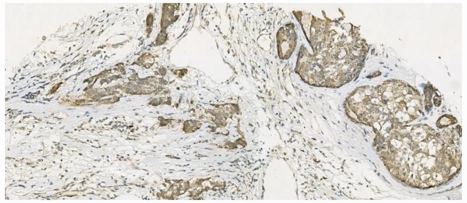
D4
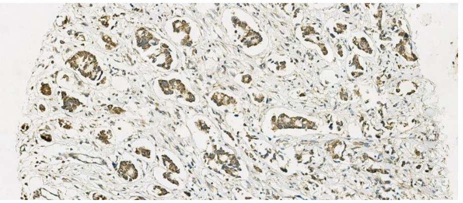
D5
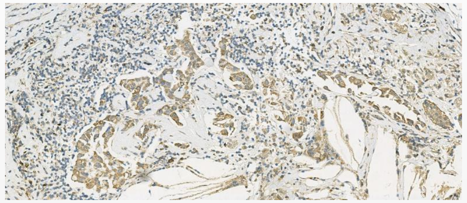
D6
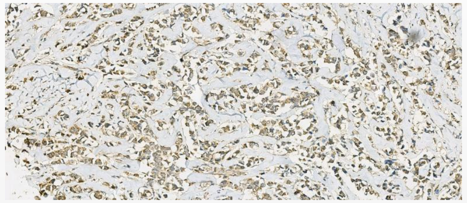
D7
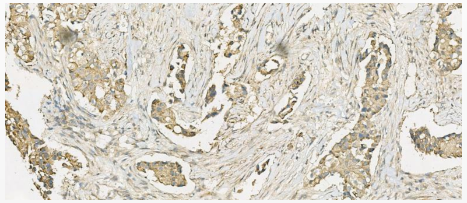
D8
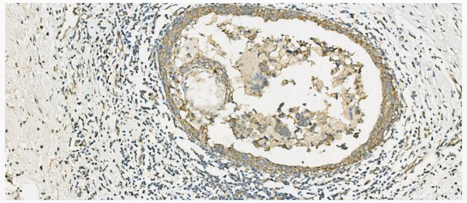
D9
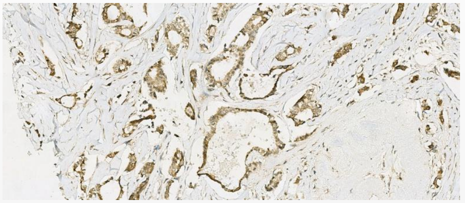
D10
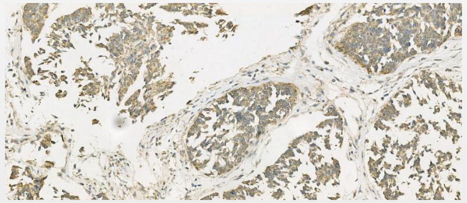
D11
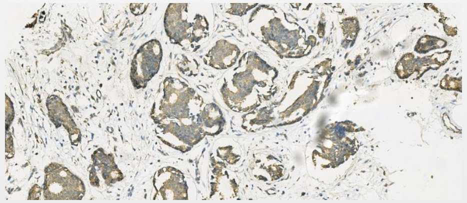
D12
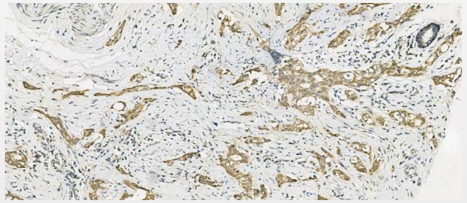
D13
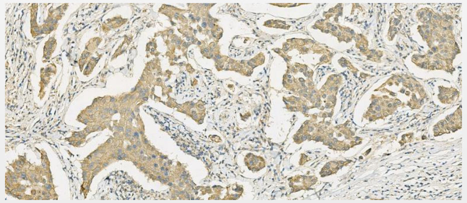
D14
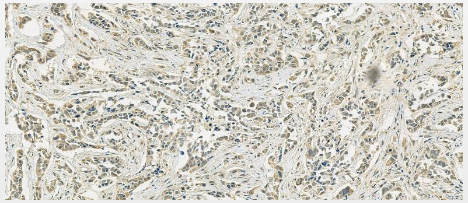
D15
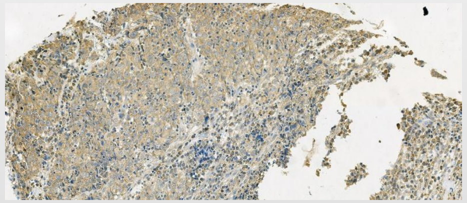
D16

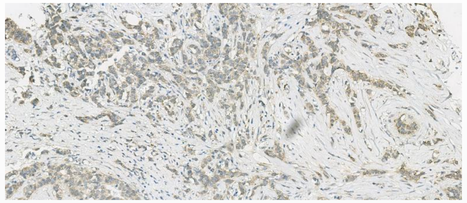
E1

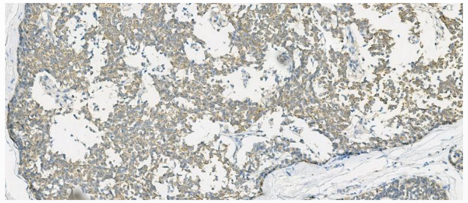
E2

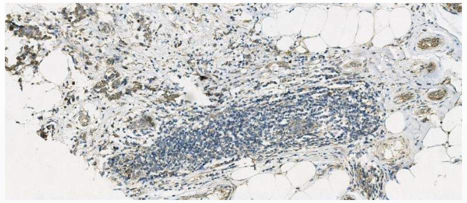
E3

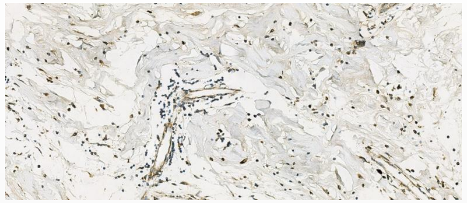
E4

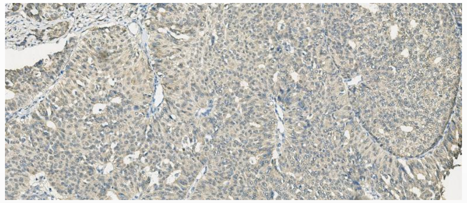
E5

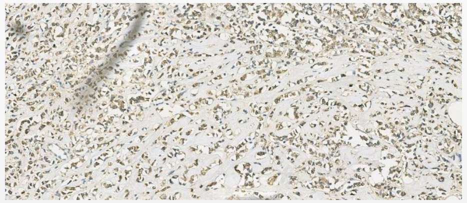
E6

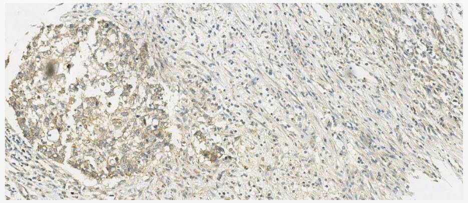
E7

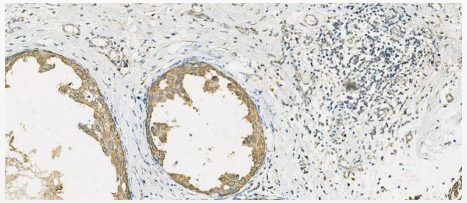
E8

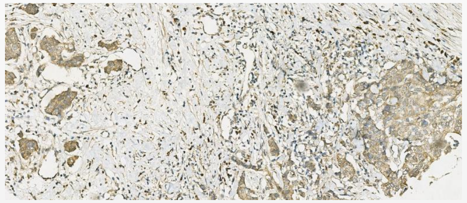
E9

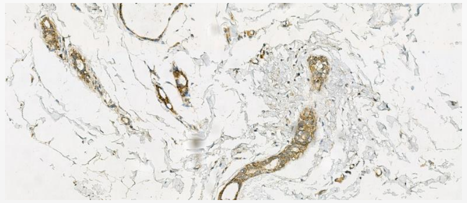
E10

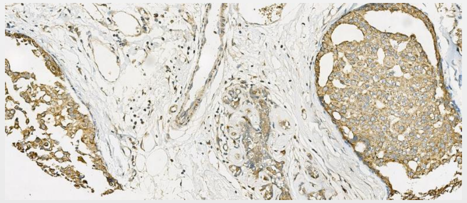
E11

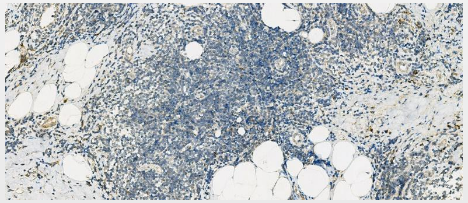
E12

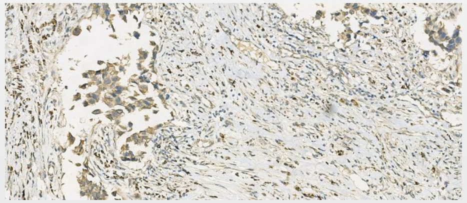
E13

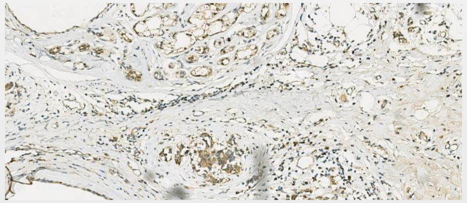
E14

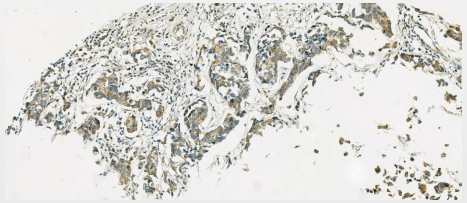
E15

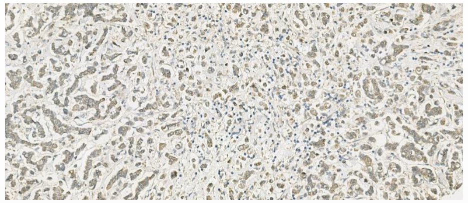
F2

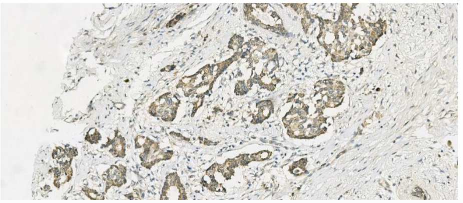
F3

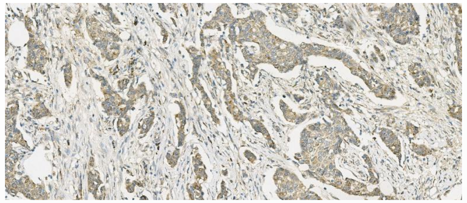
F4

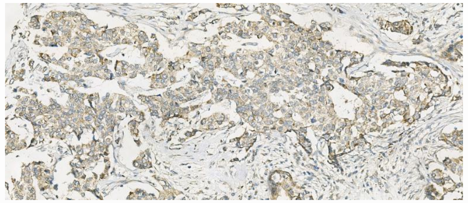
F5

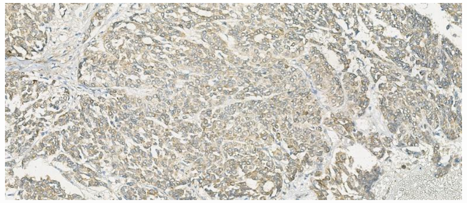
F6

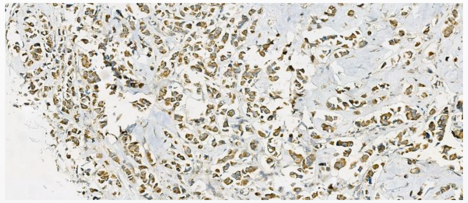
F8

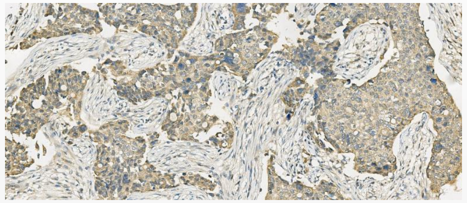
F9

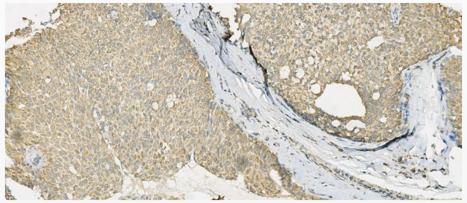
F10

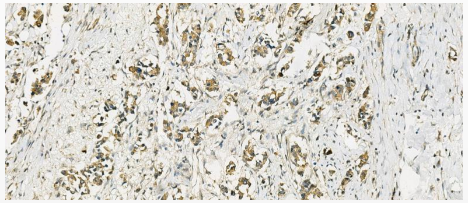
F11

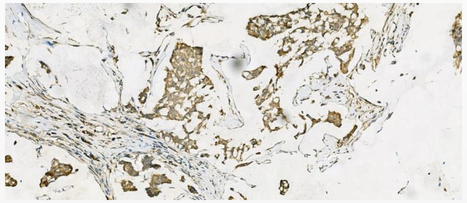
F12

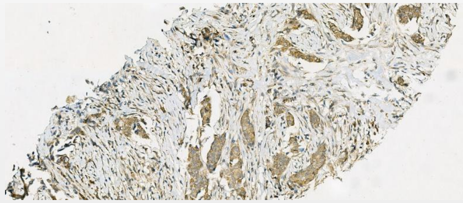
F13

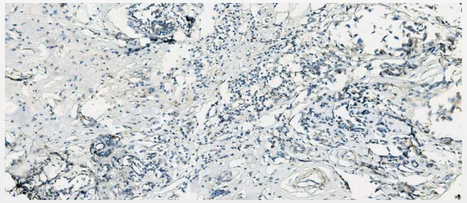
F14

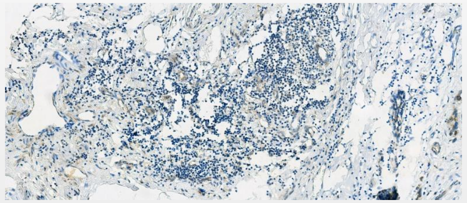
F15

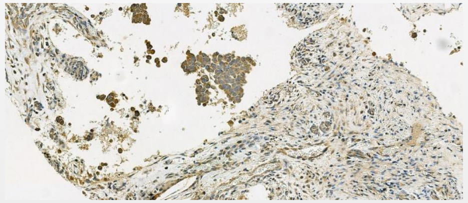
F16

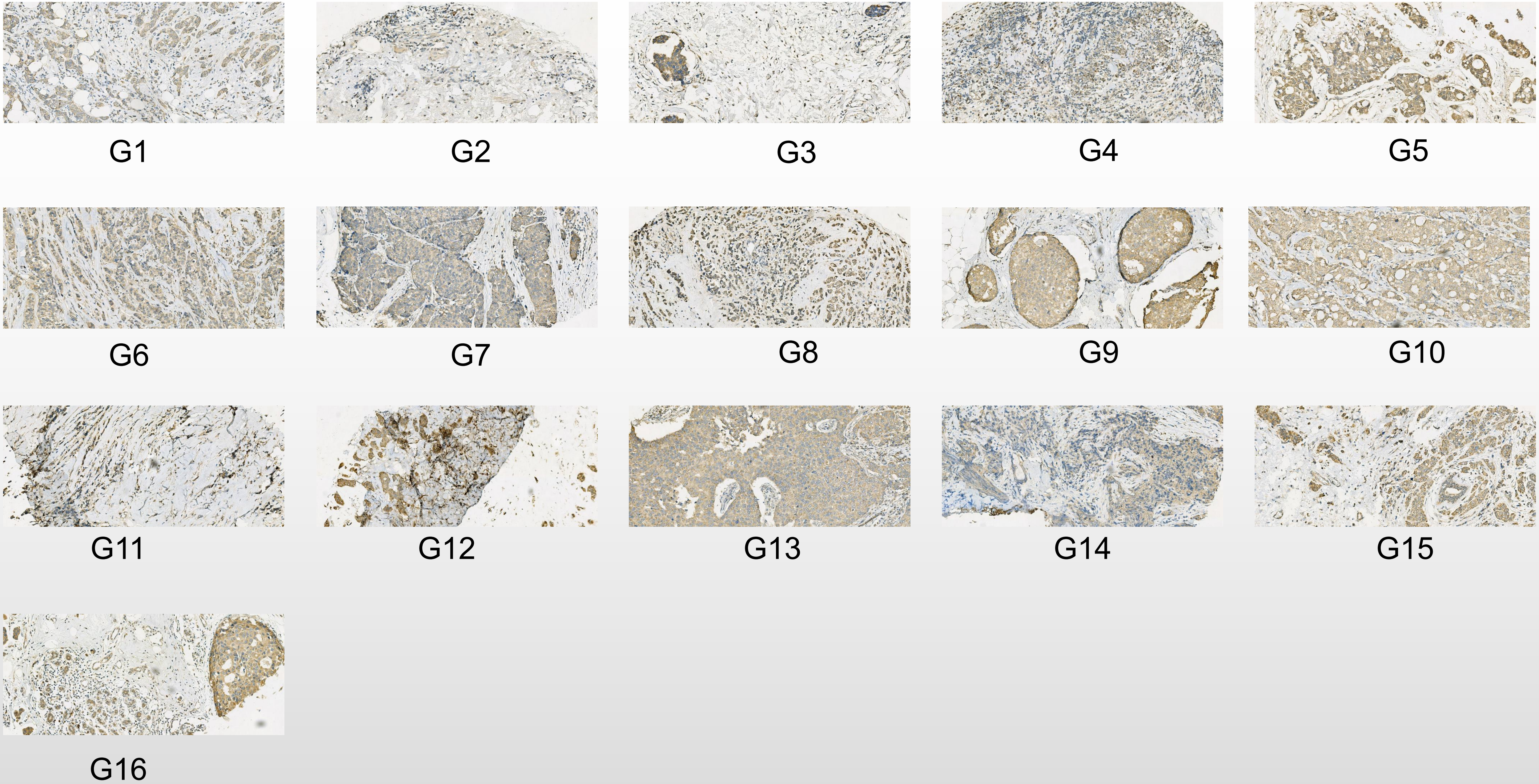
G1
G2
G3
G4
G5
G6
G7
G8
G9
G10
G11
G12
G13
G14
G15
G16

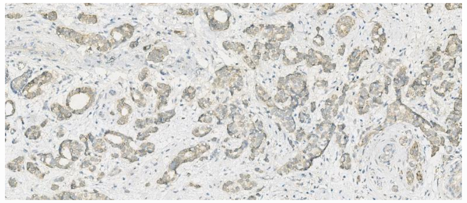
I1

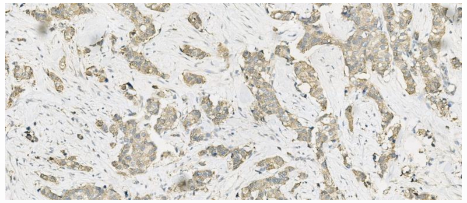
I2

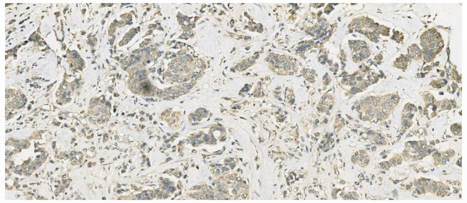
I3

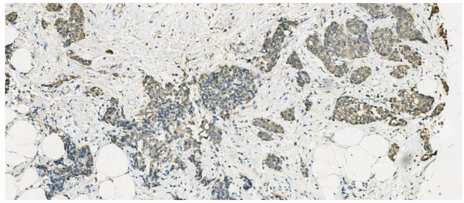
I4

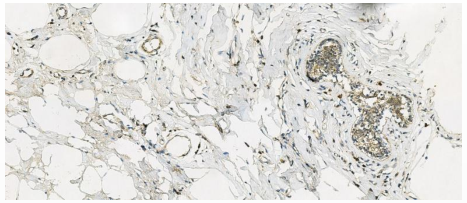
I5

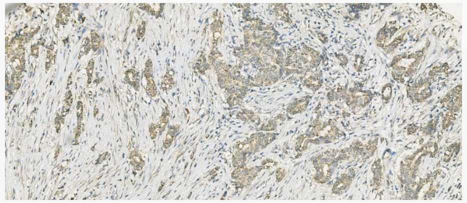
I6

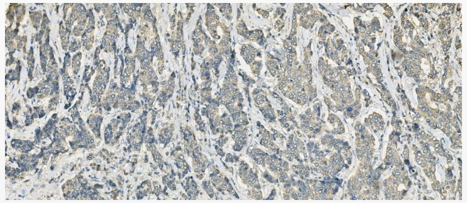
I7

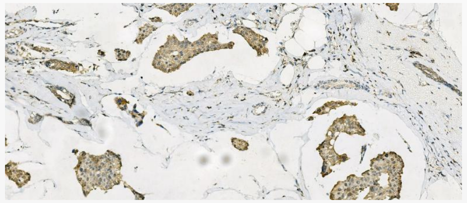
I8

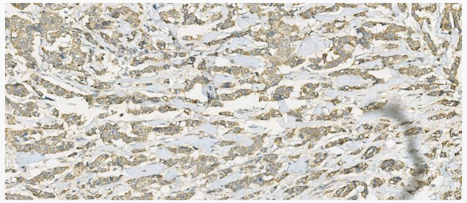
I9

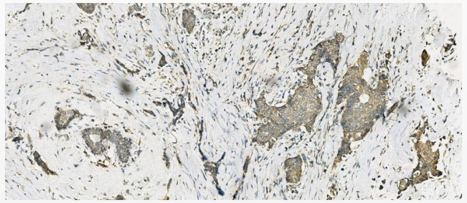
I10

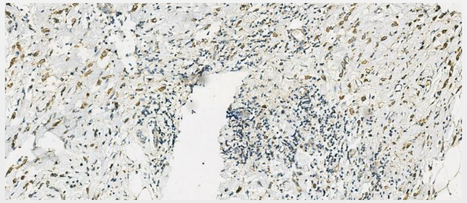
I11

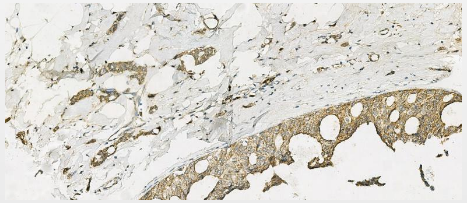
I12
